# Serial Tumor-Agnostic Plasma Profiling with a Compact NGS Panel During First-Line Therapy for Newly Diagnosed Metastatic Colorectal Cancer

**DOI:** 10.3390/ijms27219633

**Published:** 2026-10-28

**Authors:** Galina Zakharova, Oleg Zauzolkov, Anna Khristichenko, Vladimir Lyadov, Ivan Gaziev, Nina Shaban, Anna Emelyanova, Marina Sekacheva, Marina Lyadova, Anton Buzdin

**Affiliations:** 1 Institute of Personalized Oncology, Sechenov First Moscow State Medical University ( Sechenov University), 119991 Moscow, Russia; gaziev_i_r@staff.sechenov.ru (I.G.); shaban_n_a@staff.sechenov.ru (N.S.); emelyanova_a_a@staff.sechenov.ru (A.E.); sekacheva_m_i@staff.sechenov.ru (M.S.); 2 City Clinical Hospital Named After S.S. Yudin, Moscow Healthcare Department, 129090 Moscow, Russia; mir1989@inbox.ru (O.Z.); vlyadov@gmail.com (V.L.); dr.lyadova@gmail.com (M.L.); 3 Center of Genetics and Life Sciences, Sirius University of Science and Technology, 354340 Sirius Federal Territory, Russia; anya.khristichenko@gmail.com; 4 Department of Oncology and Palliative Medicine Named After Academician A.I. Savitsky, Russian Medical Academy of Continuous Professional Education, 125993 Moscow, Russia

**Keywords:** liquid biopsy, metastatic colorectal cancer, cell-free DNA, cfDNA, circulating tumor DNA, ctDNA, oncogenic mutations, molecular diagnostics, NGS

## Abstract

Plasma circulating tumor DNA (ctDNA) profiling may complement tissue hotspot testing and reveal molecular changes during treatment of metastatic colorectal cancer (mCRC). We prospectively studied 39 patients with newly diagnosed unresectable mCRC receiving first-line therapy. Cell-free DNA was collected before treatment and after two and four cycles and analyzed using a 10 kb, 10-gene hybrid-capture sequencing panel with molecular barcodes and error correction. Baseline oncogenic variants were detected in 32 of 35 evaluable patients (91.4%). Compared with routine tissue PCR for shared *RAS*/*BRAF* targets, plasma testing showed 84.2% sensitivity (16/19) and 100% specificity (19/19) in the intention-to-test cohort, compared with 88.2% sensitivity (15/17) and 100% specificity (17/17) among technically evaluable samples. Three activating *KRAS* variants (L19F, A59T, and A146T) lay outside the tissue assays’ target regions; patients carrying these alterations received anti-EGFR therapy. Patients with metastases in two or more organs had more detectable oncogenic alterations than those with one metastatic site (median, three versus two; adjusted *q* = 0.00184; rank-biserial correlation = 0.687). Serial sampling revealed divergent trajectories of individual variants in nine patients. Early reductions in plasma variant allele frequencies tended to be greater among partial responders, although associations with radiological response were not significant after multiple testing correction. Compact tumor-agnostic plasma sequencing provides complementary molecular information, but its clinical utility requires further validation.

## 1. Introduction

Metastatic colorectal cancer (mCRC) is a molecularly heterogeneous disease, and molecular testing is an integral part of treatment planning. In particular, *KRAS* and *NRAS* status is required when considering anti-epidermal growth factor receptor (anti-EGFR) therapy, while *BRAF* V600E and mismatch repair status also inform molecular characterization and treatment selection [1,2]. Tissue remains the standard source for tumor genotyping, but a tissue result reflects the sampled lesion at a particular time point and may not capture the full molecular diversity of metastatic disease.

Plasma circulating tumor DNA (ctDNA) provides a minimally invasive source of tumor-associated molecular information that can be sampled repeatedly. Prospective studies have shown that plasma testing can detect *RAS* mutations in mCRC and can achieve substantial agreement with tissue-based results [3,4,5]. Large clinical studies have further evaluated plasma genotyping in routine oncology settings [6]. However, plasma and tissue results may be discordant. Low ctDNA shedding can lead to false-negative plasma findings, while spatial or temporal heterogeneity and differences in assay coverage may also contribute to discordance [3,4,5,7]. The interpretation of plasma testing, therefore, depends on both the amount of ctDNA present and the genomic regions interrogated by the assay.

The amount of ctDNA detected in plasma may vary with metastatic burden and the anatomical distribution of disease [8,9]. This variability is relevant when interpreting a negative plasma result and when comparing ctDNA profiles between patients. Serial sampling may partly address the limitations of a single measurement by showing how circulating molecular signals change during treatment.

In mCRC, early reductions in ctDNA during systemic therapy have been associated with subsequent treatment response, and prospective studies have evaluated ctDNA kinetics during first line treatment [10,11,12,13]. Beyond changes in overall ctDNA burden, longitudinal sequencing can reveal distinct trajectories among individual variants, including persistence, disappearance, or emergence during therapy [14]. Such differences may reflect the molecular heterogeneity of metastatic disease and treatment-associated changes in tumor subclones. Nevertheless, using early ctDNA dynamics to guide treatment or predict response remains an area under investigation rather than an established routine application [7,15].

A compact, tumor-agnostic targeted panel could enable serial profiling of recurrently altered colorectal cancer genes without requiring individualized assay design based on prior sequencing of each patient’s tumor. It may also identify plasma variants outside the target regions of a routine tissue hotspot assay. Such findings do not establish that the tissue assay produced an analytical false negative; rather, they show that assays with different target coverage can provide different molecular information. The potential contribution of a compact panel should, therefore, be assessed alongside its coverage, plasma–tissue agreement for assessable variants, and performance across serial samples.

In this prospective study, we evaluated a compact, error-corrected, tumor-agnostic targeted sequencing assay in patients with newly diagnosed, unresectable mCRC receiving first line systemic therapy. We characterized baseline plasma alterations and compared plasma detection of assessable *RAS*/*BRAF* targets with routine tissue-based polymerase chain reaction (PCR). We also recorded plasma alterations outside the comparator assay’s target coverage and examined changes in variant allele frequencies (VAFs) after two cycles and after four cycles of therapy, at the first radiological assessment. Particular attention was given to whether individual variants followed concordant or divergent trajectories. Finally, we explored associations between plasma findings, metastatic burden, and subsequent clinical management, including consideration for surgery. Because plasma results were not available to treating clinicians, the study describes assay feasibility and molecular patterns; it does not evaluate whether cfDNA-guided decisions improve treatment or patient outcomes.

## 2. Results

### 2.1. Patient Cohort and Sample Availability

**Figure 1:**
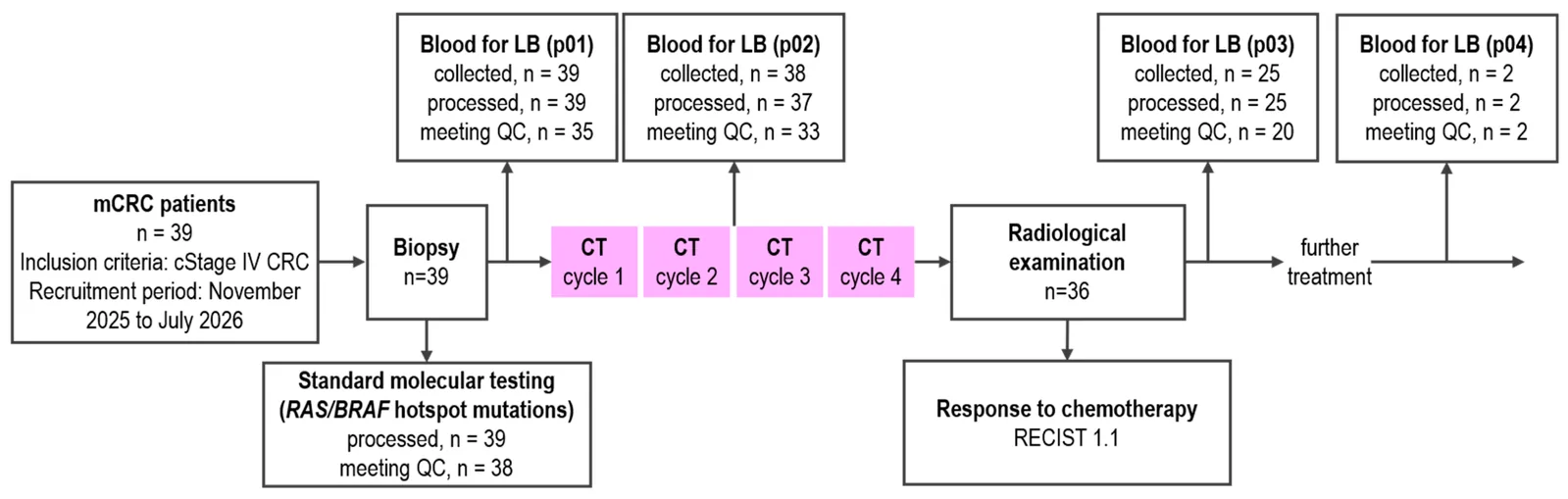
Study design and patient enrollment outline. mCRC, metastatic colorectal cancer; LB, liquid biopsy; QC, quality criteria; CT, chemotherapy; RECIST, Response Evaluation Criteria in Solid Tumors.

Thirty-nine patients with newly diagnosed unresectable stage IV colorectal cancer were prospectively enrolled (Figure 1). Median age was 66 years (interquartile range [IQR], 59–70.5; range, 44–77), and the cohort included 20 men and 19 women. The primary tumor was located in the sigmoid colon in 14 patients and in the rectum in 14; the remaining patients had tumors in other colorectal sites (Table 1). All patients received first-line systemic therapy: 14 received FOLFOX (leucovorin, fluorouracil, and oxaliplatin), 13 received chemotherapy combined with bevacizumab, and 12 received chemotherapy combined with an anti-EGFR agent. In four patients (LB_UC24, LB_UC25, LB_UC27, and LB_UC40), the regimen was modified after the first two cycles and before the first radiological assessment. One patient (LB_UC34) discontinued study participation before the p02 blood draw, while another patient (LB_UC11) discontinued immediately after p02.

Blood was collected at three time points: before therapy (p01), after two cycles (p02), and after four cycles (p03), at the time of planned first radiological assessment. Of 104 collected plasma samples, 103 were processed and one sample was excluded because of severe hemolysis. Twelve processed samples had consensus-read coverage below the prespecified 1000× threshold for reliable detection of low-VAF variants. These samples were excluded only from analyses requiring high sequencing coverage.

During follow-up, disease progression was recorded in 12 of 39 patients (30.8%). Among these patients, median time to progression was 5.4 months (IQR, 4.1–6.4 months; range, 2.2–7.5 months).

### 2.2. Baseline Plasma Profile

**Figure 2:**
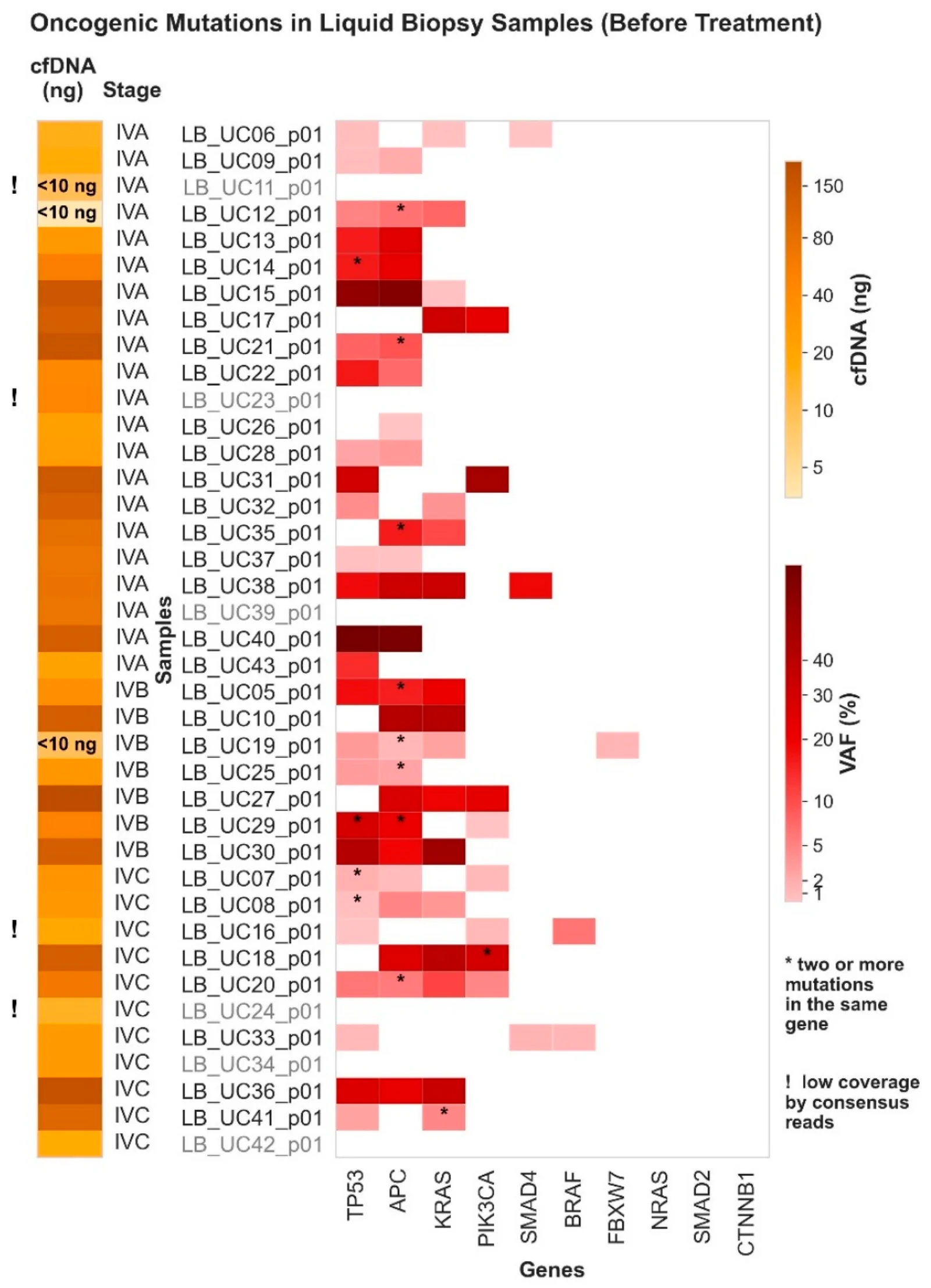
Distribution of oncogenic somatic mutations across genes and samples identified in baseline cell-free DNA (cfDNA) samples (p01). Samples for which no oncogenic somatic mutations were detected are shown in gray font. VAF, variant allele frequency.

The targeted panel covered selected exons in ten colorectal cancer-associated genes: *KRAS*, *NRAS*, *BRAF*, *APC*, *TP53*, *PIK3CA*, *FBXW7*, *SMAD2*, *SMAD4*, and *CTNNB1* (Table S1). At baseline, 35 of 39 patients had consensus-read coverage above the prespecified threshold (Table S2). Oncogenic alterations were detected in 32 of these 35 patients (91.4%; Table S3). The median number of alterations per patient was three (IQR, 2–3; range, 0–6); 31 patients had at least two detected alterations. The most frequently affected genes were *APC, TP53, KRAS,* and *PIK3CA* (Figure 2 and Figure S4).

Median plasma cfDNA concentration at p01 was 12.2 ng/mL (IQR, 7.3–22.0; range, 2.2–930.2). Among the baseline plasma profiles, median maximum VAF (maxVAF) was 11.1% (IQR, 2.54–30.2%; range, 0–79.2%), and median aggregated VAF (aggVAF) was 23.93% (IQR, 5.70–69.15%; range, 0–153.5%). The aggVAF represents the sum of the VAFs of detected alterations and may exceed 100%.

### 2.3. Associations with Primary Tumor Location and Metastatic Burden

Baseline liquid biopsy parameters including cfDNA concentration, number of detected oncogenic alterations, maxVAF, and aggVAF, did not differ significantly by primary tumor location (Figure S1).

We then assessed whether baseline plasma measurements were associated with the presence of metastases at individual anatomical sites. Comparisons were performed for liver, lung, peritoneal, and lymph-node metastases (Figure S2). None of the site-specific comparisons remained statistically significant after Benjamini–Hochberg correction. Patients with liver metastases showed nominal differences in maxVAF (*p* = 0.0173) and aggVAF (*p* = 0.0133), but both comparisons had an adjusted *q* value of 0.092. The group without liver metastases was small (*n* = 5). A nominal difference in the number of alterations between patients with and without lung metastases also did not remain significant after correction (*p* = 0.0146; *q* = 0.092).

In contrast, the number of metastatic sites was associated with the number of alterations detected in plasma. Patients with metastases in two or more organs had more detectable oncogenic alterations than those with a single metastatic site (median, 3 vs. 2; *p* = 0.00046; Benjamini–Hochberg-adjusted *q* = 0.00184; rank-biserial correlation = 0.687, 95% CI, 0.422–0.891; Figure S3). The other measured liquid biopsy parameters did not show a statistically significant difference between these groups.

### 2.4. Comparison of Plasma Findings with Routine Tissue PCR

Matched tissue-based results from routine PCR assays for *KRAS, NRAS,* and *BRAF* were available for 38 patients (Table 2 and Tables S2 and S3).

Three tissue-detected *RAS* alterations were not detected in the corresponding baseline plasma sample (Table 2). In one patient (LB_UC11), the plasma sample contained less than 10 ng of extracted cfDNA and had low consensus-read coverage. In LB_UC26, the tissue-detected *NRAS* G12C alteration was absent at p01 but became detectable at p02 and p03, with VAFs of 1.52% and 0.36%, respectively.

Plasma sequencing also identified three *KRAS* alterations outside the target coverage of the corresponding routine tissue PCR assay, among them rare activating mutations *KRAS* L19F, *KRAS* A59T, and *KRAS* A146T. Two of these alterations (*KRAS* L19F and *KRAS* A59T) were not detectable by the tissue PCR assay because it targeted only predefined *RAS*/*BRAF* hotspot mutations; both patients received anti-EGFR therapy based on the tissue results. A third plasma *KRAS* A146T alteration was also outside the target coverage of the tissue PCR assay; although *KRAS* exon 4 may be included in some tissue-based assays, it was not assessed by the assay used for this patient (LB_UC36). These three variants were not included in the sensitivity and specificity calculations because the comparator PCR assay did not interrogate them.

Additional plasma variants were observed in some patients with tissue–plasma agreement for the tested hotspot, including a second *KRAS* alteration in one patient (LB_UC41).

In the full intention-to-test cohort, among the *RAS*/*BRAF* targets assessable by both approaches, plasma testing showed 84.2% sensitivity (16/19; 95% CI, 62.4–94.5%) and 100% specificity (19/19; 95% CI, 83.2–100%) relative to tissue PCR. The corresponding positive and negative predictive values (PPV and NPV) were 100% (16/16; 95% CI,80.6–100%) and 86.4% (19/22; 95% CI, 66.7–95.3%), respectively.

Among the 34 patients whose baseline liquid biopsy results met the prespecified sequencing-depth criterion (consensus sequencing depth ≥1000×; LB_UC11, LB_UC16, LB_UC23, and LB_UC24 excluded; Table S2), plasma testing showed 88.2% sensitivity (15/17; 95% CI, 65.7–96.7%) and 100% specificity (17/17; 95% CI, 81.6–100%) relative to tissue PCR. The corresponding PPV and NPV were 100% (15/15; 95% CI, 79.6–100%) and 89.5% (17/19; 95% CI, 68.6–97.1%), respectively.

### 2.5. Longitudinal Changes in Plasma-Detected Variants During First-Line Therapy

**Figure 3:**
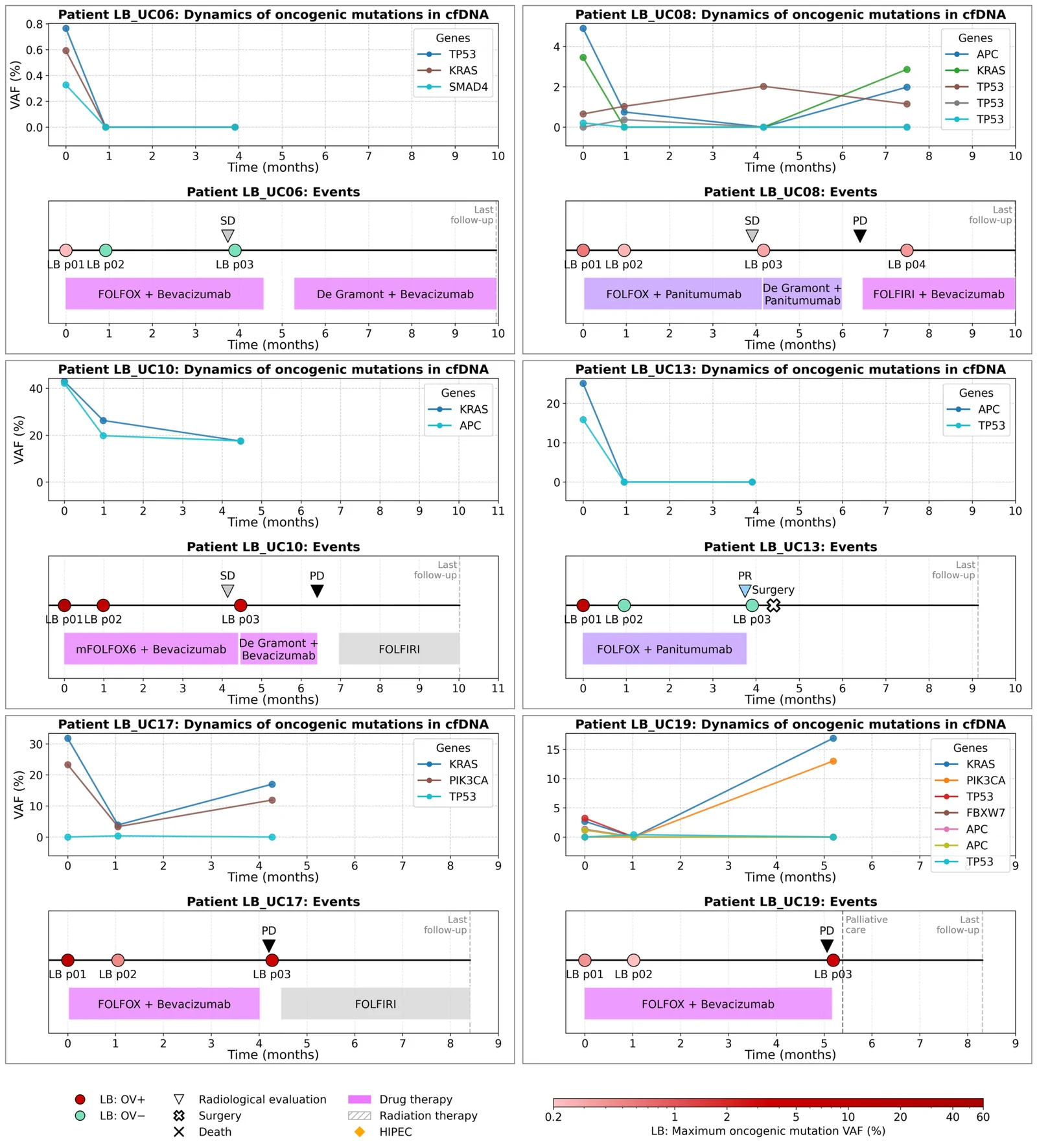
Longitudinal dynamics of oncogenic somatic mutation variant allele frequencies (VAFs) in cfDNA and treatment course and radiological response in individual patients with mCRC. LB, liquid biopsy; OV, oncogenic variants; HIPEC, hyperthermic intraperitoneal chemotherapy; CR, complete response; PR, partial response; SD, stable disease; PD, progressive disease.

At p02, 33 patients had paired baseline and post-two-cycle plasma profiles with sufficient sequencing coverage for evaluation (Table S2). Oncogenic alterations were detectable at both time points in 22 patients (66.7%) (Table S3). In seven patients (21.2%), baseline alterations were not detected in the p02 sample; four patients had no detectable alterations at either time point. Individual plasma trajectories are shown together with treatment course and radiological response in Figure 3 and Figures S5–S11.

Across the cohort, VAFs generally decreased after two cycles of treatment (Figure 3 and Figures S5–S11). Two patients, LB_UC26 and LB_UC38, showed increases in the VAFs of all detected oncogenic alterations at p02. In other patients, the direction of change differed between variants within the same plasma profile. For example, in LB_UC07, the VAFs of two of four detected alterations increased while those of the other two decreased. In LB_UC41, two baseline alterations in *KRAS* and *TP53* were no longer detected at p02, whereas another *KRAS* alteration became detectable.

Across p01–p03, divergent trajectories among individual variants were observed in nine patients: LB_UC07, LB_UC08, LB_UC12, LB_UC17, LB_UC19, LB_UC25, LB_UC26, LB_UC29, and LB_UC41 (Figure 3 and Figures S5–S11). The profiles included variants that persisted or increased while other variants in the same patient decreased or became undetectable, as well as alterations that became detectable at a later sampling point.

Two patients illustrate different patterns in more detail. In LB_UC08, five oncogenic alterations were detected across the sampling points, including three *TP53* alterations, with discordant VAF patterns. This patient had metastases involving the lungs, peritoneal cavity, lymph nodes, and pleura (Figure 3). In LB_UC19, five alterations in *KRAS, APC, TP53,* and *FBXW7* were detected at p01 at VAFs of approximately 1–3%. These baseline alterations were not detected at p02, when a newly detected *TP53* variant was observed at approximately 0.4%. By p03, the VAF of the baseline *KRAS* alteration had increased to 17%, and a *PIK3CA* variant was detected at up to 13% (Figure 3).

### 2.6. Liquid-Biopsy Findings in Patients Considered for Surgery

Following the first radiological assessment after four cycles of therapy, 12 of 39 patients (30.8%) were considered eligible for surgery (Table S4). Among these 12 patients, no oncogenic variants were detected in the last available preoperative plasma sample in six (Table S3). Three additional patients had a maxVAF below 1%. In the remaining three patients, maxVAF exceeded 1%: 2.6% at p02 in LB_UC14, 1.4% at p03 in LB_UC21, and 9.4% at p03 in LB_UC38.

**Figure 4:**
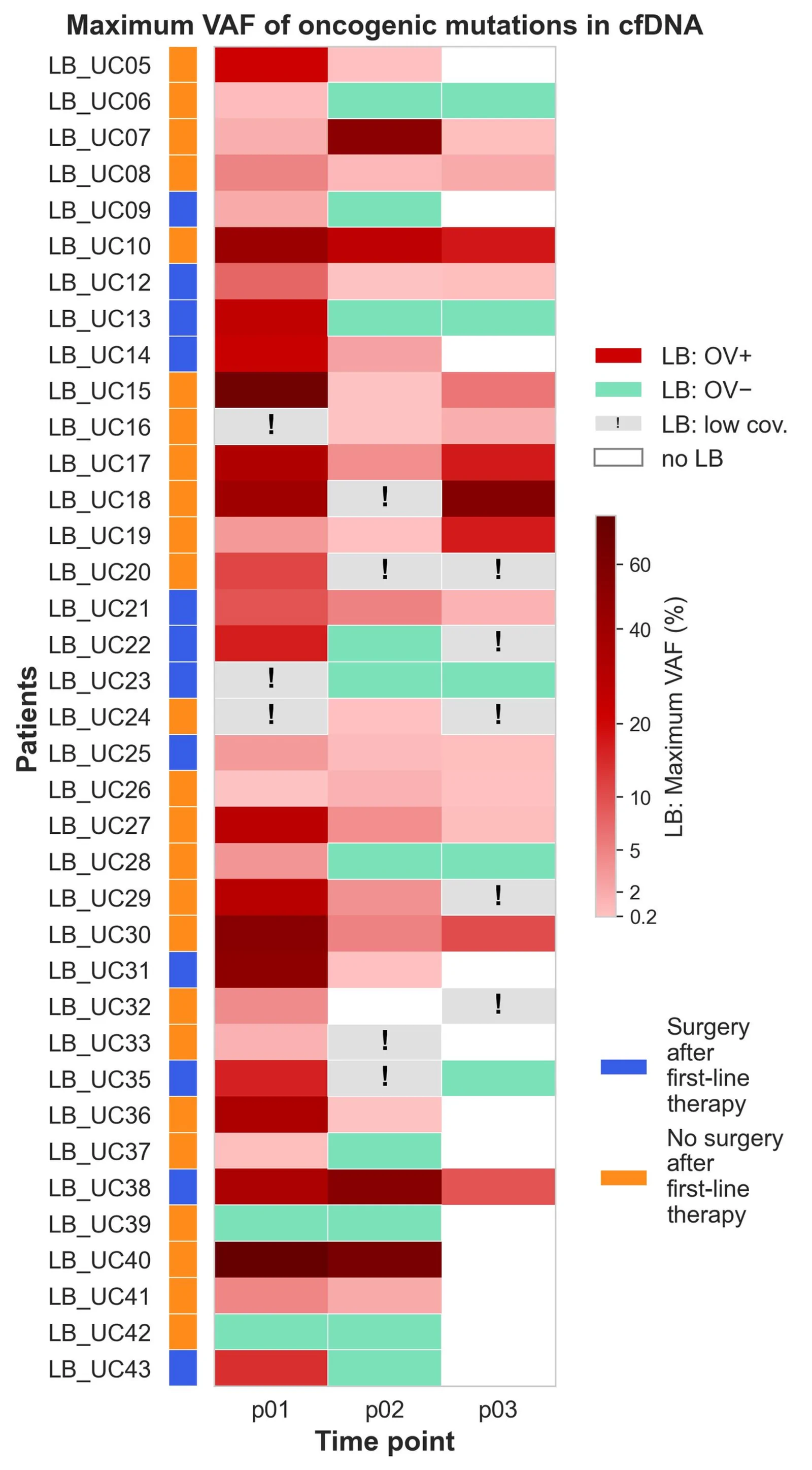
Maximum variant allele frequency (VAF) of oncogenic mutations in cell-free DNA (cfDNA) at three time points (p01, p02, p03) in patients with metastatic colorectal cancer. LB, liquid biopsy; OV, oncogenic variants. Patients shown in blue were considered resectable after first-line systemic therapy and subsequently underwent surgery; patients shown in orange did not undergo surgery.

Many patients who did not undergo surgery had higher VAFs of plasma-detected oncogenic variants, although some had low or undetectable levels (Figure 4 and Figure S12). Comparison of maxVAF and aggVAF, and other liquid-biopsy parameters between patients who underwent surgery and those who did not showed no statistically significant differences. The distribution of maxVAF and aggVAF at p02 and p03 in these groups is presented in Figure S13.

### 2.7. Association Between Changes in Plasma-Detected Oncogenic Variants and Radiological Response According to RECIST

To explore the relationship between early molecular changes and treatment response, we assessed the associations of maxVAF and aggVAF levels, as well as changes in these measures from baseline (p01) to after two and four treatment cycles (p02 and p03, respectively), with radiological response at the first assessment according to RECIST 1.1. Given the limited sample size, this analysis was considered exploratory.

Response data were unavailable for three of the 39 patients: LB_UC11 and LB_UC34 discontinued the study before the first radiological assessment, and the first assessment for LB_UC41 was still pending. Four additional patients (LB_UC24, LB_UC25, LB_UC27, and LB_UC40) were excluded from the analysis of associations between liquid biopsy findings and treatment response because their treatment regimens were changed before the first radiological assessment (Figures S8 and S10). Among the remaining 32 patients, the best response at the first radiological assessment was partial response (PR) in 13 patients, stable disease (SD) in 13, and progressive disease (PD) in six; no complete responses (CR) were observed (Table S4).

Patients were divided into responders, comprising those with a partial response (PR), and non-responders, comprising those with stable disease (SD) or progressive disease (PD). No statistically significant differences were observed between the groups in clinicopathological characteristics, including age, number of metastatic organs, ECOG performance status, pathological stage, primary tumor and metastasis locations, or sex.

Importantly, only technically evaluable samples were included in the analyses. For comparisons between two time points, both samples from a given patient had to meet the technical quality criteria. Information on the technical characteristics of liquid biopsy samples is provided in Table S2.

Neither maxVAF nor aggVAF differed significantly between responders and non-responders before treatment initiation (p01; Figure S14). At baseline, median maxVAF was 16.30% in responders and 7.09% in non-responders (*p* = 0.397), whereas median aggVAF was 27.85% and 15.63%, respectively (*p* = 0.611). At p02, both metrics tended to be higher in non-responders: median maxVAF was 0.10% in responders versus 1.03% in non-responders (*p* = 0.093, *q* = 0.309), and median aggVAF was 0.11% versus 2.15%, respectively (*p* = 0.103, *q* = 0.309).

We next investigated whether treatment response was more closely associated with changes in the levels of detectable oncogenic variants than with their absolute levels at individual time points. Changes in maxVAF and aggVAF from baseline (p01) to p02 and p03 were assessed as both absolute and percentage changes relative to baseline (Figure S15). At p02, the median percentage reduction in maxVAF was 99.5% in responders and 87.8% in non-responders; the corresponding reductions in aggVAF were 99.7% and 87.2%, respectively. These differences showed a trend toward greater reductions in responders but did not reach statistical significance (*p* = 0.123, *q* = 0.309 and *p* = 0.109, *q* = 0.309, respectively). Overall, percentage changes appeared more discriminative than absolute changes, although no comparison of VAF dynamics reached statistical significance after multiple-testing correction. The corresponding *p*-values were generally lower for changes between p03 and p01 than for those between p02 and p01. Notably, patients who underwent surgery after the first radiological assessment had no p03 samples available; therefore, the p03 analyses included only patients who continued systemic therapy beyond the first assessment.

## 3. Discussion

In this prospective study, a compact tumor-agnostic plasma sequencing panel detected oncogenic alterations in most patients with adequate baseline sequencing coverage and identified several *KRAS* variants outside the target regions of routine tissue polymerase chain reaction (PCR). Serial profiling also showed that individual variants did not always follow the same trajectory during first-line therapy. The findings support the feasibility of combining baseline molecular profiling with variant-level monitoring in a single plasma assay. They do not establish that this approach improves treatment selection or patient outcomes.

The comparison with tissue testing needs to be interpreted according to the coverage of the comparator. In the full intention-to-test cohort, plasma testing showed 84.2% sensitivity (16/19; 95% confidence interval [CI], 62.4–94.5%) and 100% specificity (19/19; 95% CI, 83.2–100%) for the assessable *RAS/BRAF* targets. Among technically evaluable samples, sensitivity was 88.2% (15/17; 95% CI, 65.7–96.7%) and specificity was 100% (17/17; 95% CI, 81.6–100%). However, the confidence intervals were wide, reflecting the small number of evaluable cases. The three additional plasma *KRAS* variants were outside the target coverage of the routine tissue PCR assay and, therefore, were not analytical false negatives of that assay. Rather, they illustrate how a panel covering additional regions may return information that a predefined hotspot test does not provide. This distinction is important when interpreting plasma–tissue agreement: concordance can only be assessed for genomic positions measured by both methods.

Previous studies have also reported substantial agreement between plasma and tissue *RAS* testing in mCRC. In mCRC cohorts specifically evaluating *RAS* mutational status, Bando et al. reported an overall concordance of 86.4%, with a sensitivity of 82.1% and specificity of 90.4% [3], whereas Vidal et al. reported 93% overall concordance [5]. Studies employing broader molecular profiling approaches have reported different agreement rates. In patients with newly diagnosed mCRC, comprehensive ctDNA genotyping demonstrated 83.2% overall agreement with standard tissue testing across guideline-recommended biomarkers, including *KRAS*, *NRAS*, *BRAF* V600E, microsatellite instability and *ERBB2* alterations [16]. Another study involving patients with CRC at different stages reported 73.53% overall concordance between tissue and plasma NGS across a broader set of cancer-related genes [17].

Three patients with plasma *KRAS* variants outside tissue-PCR coverage received anti-EGFR therapy based on their negative tissue PCR results. All three variants (*KRAS* L19F, A59T, and A146T) have demonstrated activating effects in experimental cell line models [18,19,20,21,22]. Furthermore, OncoKB annotates *KRAS* L19F, A59T, and A146T as level R1 for resistance to cetuximab and panitumumab in colorectal cancer. In our cohort, the patient with *KRAS* L19F who received panitumumab had stable disease (SD) at the first radiological assessment, followed by progressive disease (PD) at the second assessment (Figure S5), whereas the patient with *KRAS* A59T who received cetuximab initially achieved a partial response (PR) but experienced PD shortly thereafter (Figure S6). In contrast, the patient with *KRAS* A146T also achieved a PR during anti-EGFR therapy (Figure S10). Among nine additional patients who received anti-EGFR therapy and had no *RAS*/*BRAF* alterations detected by either tissue PCR or plasma cfDNA profiling, seven achieved a partial response (PR) at the first radiological assessment, one had stable disease (SD), and one had progressive disease (PD) (Figures S5–S11). Four of the seven patients with a PR were subsequently considered eligible for surgery and underwent resection. These individual observations do not establish a causal relationship between any of the additional *KRAS* variants and treatment response or resistance. Nevertheless, they illustrate the potential relevance of detecting activating *KRAS* variants outside the regions assessed by routine tissue assays and raise the possibility that broader plasma profiling could identify additional potentially treatment-relevant alterations. The predictive significance of these findings requires investigation in larger cohorts.

In our cohort, baseline plasma profiling detected oncogenic alterations in 91.4% (32 of 35) of mCRC patients with technically adequate sequencing coverage, with most patients harboring multiple alterations and *APC*, *TP53*, *KRAS*, and *PIK3CA* being the most frequently affected genes. Previous studies have reported baseline plasma mutation detection rates ranging from 76.8% to 95.7% in patients with mCRC, depending on the assay and molecular targets. Using the Safe-SeqS assay, patient-specific candidate mutations identified in tumor tissue were detectable in plasma cfDNA in 48 of 52 patients (92.3%) [10]. In another cohort, baseline ctDNA was detected in 63 of 82 patients (76.8%) using mutation-targeted or methylation-based picodroplet-digital PCR assays, with no significant difference in detection rates between the two approaches [23]. Among 43 patients with tissue-confirmed *KRAS* (*n* = 37), *TP53* (*n* = 3), or *BRAF* (*n* = 3) mutations, the corresponding mutation was detected in baseline plasma in 36 (83.7%) [23]. Using amplicon-based NGS targeting 14 genes commonly mutated in colorectal cancer, somatic mutations, including single-nucleotide variants and short insertions and deletions, were identified in plasma cfDNA in 88 of 101 mCRC patients (87.1%) [24]. A similar detection rate was reported in patients receiving second-line chemotherapy, in whom the same panel detected at least one somatic mutation in 26 of 29 patients (89.7%) [25]. Finally, NGS profiling of 50 cancer-related genes using the Ion Proton platform detected mutations in 95.7% of pretreatment ctDNA samples from patients receiving first-line chemotherapy [11].

A further consideration is the origin of plasma variants. Because matched leukocyte DNA was not sequenced, variants arising from clonal hematopoiesis cannot be completely excluded, particularly for genes such as *TP53*. High-intensity sequencing studies have shown that clonal hematopoiesis can contribute substantially to plasma cfDNA variants and that matched white blood cell sequencing can help identify their source [26]. Accordingly, the plasma-only variants in this study should be interpreted as alterations detected in cfDNA rather than definitively proven tumor-derived variants unless supported by tissue findings or other evidence.

The observed decrease in VAFs in most patients after two treatment cycles is consistent with prospective mCRC studies in which early ctDNA decline was associated with treatment response [10,13]. Other longitudinal work has shown that ctDNA profiles can change during treatment and may provide information beyond a single baseline measurement [12]. In our study, exploratory analyses suggested that changes in plasma variant levels may be more informative than their absolute levels for assessing treatment response. Responders (PR) showed numerically greater percentage reductions in maxVAF and aggVAF after two treatment cycles than non-responders (SD and PD), although these differences were not statistically significant after multiple-testing correction. At p03, differences in absolute VAF levels were more pronounced but likewise did not remain significant after correction. Importantly, the p03 analysis included only patients who had not undergone surgery after the first radiological assessment and continued systemic therapy. These findings suggest a potential association between plasma variant dynamics and radiological response; however, the small comparison groups substantially constrain interpretation. The results should therefore be considered hypothesis-generating and require validation in larger prospective cohorts.

Interestingly, in our study, one patient (LB_UC07) showed a sharp increase in the VAFs of plasma-detected oncogenic variants at p02 (after two treatment cycles), followed by a decline to very low levels at p03 (after four treatment cycles). Similar transient ctDNA spikes have been reported elsewhere. For example, in study [10], four of 53 patients with mCRC exhibited a sharp increase in ctDNA levels 3 days after chemotherapy initiation, followed by a decline at the next time point (before cycle 2). The authors hypothesized that, because three of these patients had an excellent response (≥20% reduction in tumor size at the first restaging assessment), the transient spike might reflect rapid tumor DNA release into the circulation from treatment-responsive tumors. An important observation in this cohort was that variants within a patient could show divergent VAF trajectories. Across p01–p03, such patterns were seen in nine patients; some variants decreased or became undetectable while others persisted, increased, or became detectable at a later time point. Even when *TP53* variants, which, as mentioned above, cannot be distinguished from variants arising from clonal hematopoiesis in the absence of matched leukocyte sequencing, are excluded from consideration, four patients (LB_UC07, LB_UC19, LB_UC26, and LB_UC41) still showed divergent trajectories of plasma variants. This illustrates why the change in one mutation—or in an aggregate measure such as maxVAF—may not describe every variant in a patient’s plasma profile. Studies of mCRC treated with anti-EGFR agents have documented the emergence and subsequent decline of resistance-associated *RAS* and *EGFR* clones, and the CHRONOS trial used ctDNA to select patients for anti-EGFR rechallenge [14,15]. The present study extends the description of variant-level dynamics to a mixed first line treatment cohort, but it cannot determine whether newly detected variants represent newly emerging clones, expansion of previously low-frequency clones, or fluctuations in detectability near the assay’s reporting threshold. Because orthogonal validation and replicate sequencing were not performed, technical variability cannot be excluded, particularly for low-frequency variants. Accordingly, these observations should be interpreted as descriptive patterns of plasma variant detectability rather than definitive evidence of clonal evolution.

At baseline, patients with metastases in two or more organs had a greater number of detectable oncogenic alterations than patients with a single metastatic site. This is compatible with the possibility that a broader distribution of disease increases the repertoire of oncogenic alterations detectable in plasma. Prior mCRC studies have also reported relationships between ctDNA measurements and metastatic sites or baseline prognosis [8,9]. In this cohort, however, individual metastatic-site comparisons did not remain significant after Benjamini–Hochberg correction. The small number of patients without particular sites of metastasis, together with the absence of a direct measurement of total tumor volume, limits interpretation of the association with the number of metastatic organs.

Among patients considered eligible for surgery after the first radiological assessment, oncogenic variants were undetectable in half of the last available preoperative samples, and most of the remaining patients had low maxVAF values. Nevertheless, liquid biopsy measures did not differ significantly between patients who underwent surgery and those who did not. This exploratory comparison should not be interpreted as evidence that panel-detectable oncogenic variants in cfDNA identifies conversion to resectability. Surgical decisions depend on factors including the anatomical distribution and technical possibility to resect metastases, radiological findings, patient condition, comorbidities, and treatment preferences, and the surgical subgroup was small.

Several limitations define the scope of these findings. The study included 39 patients from a single center, and the treatment regimens were heterogeneous. The tissue comparator was routine hotspot PCR rather than comprehensive tissue NGS, preventing full assessment of plasma–tissue concordance across the panel. The study did not include matched leukocyte sequencing or independent orthogonal confirmation of plasma-only variants. Although the assay used UMI-based error correction and a prespecified VAF threshold, this cohort was not a formal analytical validation study establishing limit of detection, precision, or reproducibility. Finally, the study was not powered to assess associations between changes in plasma-detected variants and clinical outcomes, and assay results were unavailable to clinicians.

Taken together, these results support the feasibility of serial profiling with a compact tumor-agnostic panel and show that plasma analysis can provide molecular findings beyond those interrogated by routine tissue PCR. The longitudinal data also demonstrate that different variants in the same patient may follow distinct trajectories during treatment. Larger prospective studies with comprehensive paired tissue and plasma profiling, matched leukocyte sequencing, predefined molecular response criteria, and clinical outcome assessment are needed to determine the analytical and clinical utility of this approach.

## 4. Materials and Methods

### 4.1. Study Design and Participants

This single-center prospective study was conducted at Oncological Center No. 1 of the City Clinical Hospital named after S.S. Yudin (Moscow, Russia). The study design is outlined in Figure 1. The study was approved by the Local Ethics Committee (protocol No. 11.2025; approval date: 25 July 2025) and was conducted in accordance with the Declaration of Helsinki. Written informed consent was obtained from all participants before enrollment. Eligible participants were adults (≥18 years old) with newly diagnosed, unresectable clinical stage IV colorectal cancer (metastatic colorectal cancer, mCRC) who were prescribed systemic therapy. All participants completed the standard diagnostic work-up. The exclusion criteria were: previous treatment; multiple primary malignancies; other malignancies diagnosed within 5 years. Enrollment was consecutive and non-randomized, with no intervention in routine diagnostic or therapeutic management. All patients received treatment according to the clinical practice guidelines of the Ministry of Health of the Russian Federation. Between November 2025 and July 2026, a total of 39 patients with histologically confirmed colorectal adenocarcinoma were enrolled (Table 1).

Laboratory personnel were blinded to patients’ clinical and pathological data, and the resulting liquid biopsy findings were not available to treating physicians and therefore did not influence clinical management or treatment decisions.

### 4.2. Routine PCR Analysis of FFPE Tissue Samples

All patients underwent routine clinical testing of formalin-fixed, paraffin-embedded (FFPE) tumor tissue samples for hotspot mutations in *KRAS*, *NRAS*, and *BRAF*. In most cases, the tissue specimens were biopsies obtained from the primary tumor. All tissue specimens were considered adequate for analysis, with tumor cell content exceeding 30%, except for the biopsy specimen from patient LB_UC43, which was non-informative. The source and type of the analyzed tissue specimens, as well as the time interval between tissue sampling and the first blood collection for liquid biopsy, are provided in Table S2.

The same set of assays was used to analyze *KRAS*, *NRAS*, and *BRAF* mutations in all patients except LB_UC30. *KRAS* hotspot mutations were analyzed using the Test-KRAS-Tissue PCR assay (TestGen, Ulyanovsk, Russia), which detects G12D, G12A, G12R, G12V, G12S, G12C, and G13D mutations, on a CFX96 Touch instrument (Bio-Rad, Hercules, CA, USA). *NRAS* hotspot mutations were analyzed using the Test-NRAS-Tissue PCR assay (TestGen, Ulyanovsk, Russia), which detects G12D, G12C, G12S, G13D, G13R, Q61K, Q61L, and Q61R mutations, also on a CFX96 Touch instrument (Bio-Rad, Hercules, CA, USA). The *BRAF* V600E mutation was detected using the cobas 4800 BRAF V600 Mutation Test on a Cobas z480 analyzer (Roche, Basel, Switzerland).

For patient LB_UC30, *RAS* mutations in the tissue sample were analyzed using the Ras Mutation Analysis Kit (EntroGen, Woodland Hills, CA, USA) on a CFX96 Touch instrument (Bio-Rad, Hercules, CA, USA). The assay detects the following *KRAS* mutations: G12A, G12D, G12R, G12V, G12C*, G12S, G13D, Q61H, Q61L, Q61R, A59E, A59G, A59T, K117N, K117R, K117E, A146T, A146P, and A146V; and the following *NRAS* mutations: G12D, G12C, G12S, G13R, G13V, K117R, Q61H, Q61L, Q61K, Q61R, A59D, A59T, and A146T.

### 4.3. Sample Collection

Peripheral blood was collected in two 10 mL Cell-Free DNA Blood Collection Tubes (Magen, Guangzhou, China) before treatment (p01), after two cycles of systemic therapy (p02), and after four cycles of systemic therapy (p03) which coincided with the planned first radiological assessment. In two patients (LB_UC08 and LB_UC20), blood samples were collected after the second radiological assessment (p04). The total number of plasma samples was 104.

Plasma was separated within 1 h of blood collection by two sequential centrifugation steps: first at 1600× *g* for 10 min at room temperature, followed by 13,000× *g* for 10 min at 15 °C. The separated plasma was stored at −80 °C until DNA extraction.

### 4.4. Cell-Free DNA Extraction

Cell-free DNA (cfDNA) was extracted from plasma samples using the MagPure Circulating DNA Maxi Kit (Magen, Guangzhou, China), with a 60-min proteinase K incubation at 57 °C. The median plasma volume used for extraction was 8 mL (IQR, 7.9–8.0; range, 3.5–9.4 mL). The concentration of cfDNA was quantified using the Equalbit 1x dsDNA High Sensitivity Assay Kit (Vazyme, Nanjing, China) and a Qubit 4 fluorometer (Thermo Fisher Scientific, Waltham, MA, USA). Fragment size distribution was assessed using the High Sensitivity D1000 ScreenTape assay (Agilent, Santa Clara, CA, USA) on a TapeStation 4150 system (Agilent, Santa Clara, CA, USA).

### 4.5. NGS Analysis

NGS libraries from cfDNA samples were prepared using the KAPA HyperPrep Kit (Roche, Basel, Switzerland) and KAPA Universal UMI Adapter (Roche, Basel, Switzerland). Library quantification was performed using the Equalbit 1x dsDNA High Sensitivity Assay Kit (Vazyme, Nanjing, China) and a Qubit 4 fluorometer (Thermo Fisher Scientific, Waltham, MA, USA). Library size distribution was assessed using the High Sensitivity D1000 ScreenTape assay on a TapeStation 4150 system (Agilent, Santa Clara, CA, USA). Target enrichment was performed using the KAPA HyperCapture Reagent Kit and KAPA HyperCapture Bead Kit (Roche, Basel, Switzerland) and with custom hybridization oligonucleotide probes (Evrogen, Moscow, Russia) targeting regions frequently mutated in colorectal cancer (Table S1). The hybridization panel was designed to maximize coverage of colorectal cancer cases in a tumor-agnostic manner, while minimizing overall panel size. The final design of the custom hybridization panel covered approximately 10 kb of target genomic regions allowing detection of single-nucleotide variants and small insertions/deletions (indels). In this study, the term ‘tumor-agnostic’ refers to molecular profiling performed without prior knowledge of the individual patient’s tumor genotype or individualized assay design. The assay was not designed to assess copy-number alterations, gene fusions, microsatellite instability, or mismatch-repair deficiency. Sequencing was performed in paired-end mode, generating 150 bp-long paired-end reads (PE150) on a FASTASeq 300 sequencer (Genemind, Shenzhen, China). The median number of reads per cfDNA library was 3.9 million (IQR, 3.3–4.8 million; range, 1.6–8.1 million).

### 4.6. Bioinformatic Analysis of NGS Data

NGS data were processed using an automated bioinformatic pipeline comprising raw read preprocessing, generation of consensus reads, alignment, variant calling, filtering, and functional annotation. Raw FASTQ files were initially quality-filtered and trimmed using fastp (v0.23.4) [27]. Unique molecular identifier (UMI) codes were used to generate consensus reads, thereby reducing artifacts and removing duplicate reads. Consensus reads were aligned to the human reference genome hg38 (GRCh38.p14), and alignment quality metrics, including sequencing depth and target enrichment efficiency, were calculated using BWA (v0.7.19) [28], fgbio (v2.3.0) [29], GATK (v4.6.2.0) [30], SAMtools (v1.21) [31], and Picard (v3.2.0) [32] software tools. Single-nucleotide variants (SNVs) and indels were identified using LoFreq (v2.1.5) [33], with a prespecified bioinformatic reporting threshold of variant allele frequency (VAF) ≥0.15%. The 0.15% VAF reporting threshold was selected empirically based on our previous matched tissue–plasma analyses performed on the cohort of patients with resectable colorectal cancer using the same workflow. In these analyses, tumor-confirmed variants were detectable near this threshold, whereas lower-frequency signals more often represented background noise. No variant calls at or above this threshold were detected in samples from 23 healthy volunteers analyzed using the same workflow. Reported variants were additionally required to be supported by at least five alternative consensus reads. Variant processing and filtering was performed with BCFtools (v1.21) [34], and candidate variants were filtered based on sequencing depth, mapping quality, base quality, alternative allele depth, and variant allele frequency to exclude likely germline variants, variants located in problematic genomic regions, and recurrent technical artifacts. The remaining variants were annotated using the Ensembl Variant Effect Predictor (VEP) software (v115.2) [35]. Annotation included gene and transcript information, predicted functional consequences, and available clinical annotations. Population allele frequencies were annotated using MAX_AF, defined as the maximum allele frequency across populations in the 1000 Genomes Project Phase 3 [36] and the Genome Aggregation Database (gnomAD v4.1.0) [37,38] exome and genome datasets, whereas MAX_AF_POPS identifies the population(s) in which this maximum was observed. Additional allele frequency annotations were obtained from the RUSeq database, a reference dataset of the Russian population comprising 6402 exome samples retained after quality control from 7452 samples collected through three centers in Moscow and St. Petersburg [39], and recorded as RuSeq_AF. Association with oncological diseases was assessed according to the OncoKB knowledge base [40,41]. The final variant summary table was generated in Python (v3.12.5) using the NumPy, pandas, and cyvcf2 libraries.

### 4.7. Statistical Analysis

Continuous variables were summarized as median and interquartile range (IQR). Comparisons between two groups were performed using the Mann–Whitney U test, while comparisons among three or more groups were performed using the Kruskal–Wallis test followed by pairwise Mann–Whitney U tests, where appropriate. 95% confidence intervals (CIs) for diagnostic sensitivity, specificity, positive predictive value (PPV), and negative predictive value (NPV) were calculated using the Wilson score method. Samples with insufficient NGS read coverage were excluded from analyses requiring reliable variant detection. To account for multiple testing, *p*-values were adjusted using the Benjamini–Hochberg procedure to control the false discovery rate (FDR), and the resulting adjusted values are reported as *q*-values. All statistical tests were two-sided, and *p* < 0.05 was considered statistically significant; for analyses involving multiple testing, FDR-adjusted *q* < 0.05 was considered statistically significant. Statistical analyses were performed using Python 3.11 with the lifelines, SciPy, statsmodels, pandas, and NumPy libraries.

## 5. Conclusions

In this prospective cohort of patients with metastatic colorectal cancer, a compact, tumor-agnostic, error-corrected plasma sequencing panel enabled baseline and serial cfDNA profiling during first line therapy. In addition to detecting alterations covered by routine tissue PCR, plasma sequencing identified *KRAS* variants outside the PCR assay’s target regions. Longitudinal profiling also revealed divergent trajectories among individual variants, while patients with metastases in multiple organs had a greater number of detectable alterations. These findings support further evaluation of plasma profiling as a complement to tissue-based testing. Larger studies with comprehensive tissue and leukocyte comparisons, analytical validation, and clinical outcome assessment are needed to establish its clinical validity and utility.

## Figures and Tables

**Table 1:** Baseline clinicopathological characteristics of the cohort.

Characteristic	*n* (%)
**Age, years**	
Median (IQR; range)	66 (59–70.5; 44–77)
**Gender**	
Male	20 (51.3%)
Female	19 (48.7%)
**ECOG performance status**	
1	24 (61.5%)
2	15 (38.5%)
**Primary tumor location**	
Cecum	2 (5.1%)
Ascending colon	5 (12.8%)
Hepatic flexure	1 (2.6%)
Transverse colon	1 (2.6%)
Descending colon	1 (2.6%)
Sigmoid colon	14 (35.9%)
Rectosigmoid	1 (2.6%)
Rectum	14 (35.9%)
**T category**	
T2	4 (10.3%)
T3	21 (53.8%)
T4	14 (35.9%)
**N category**	
N0	6 (15.4%)
N1	19 (48.7%)
N2	14 (35.9%)
**M category**	
M1	39 (100%)
**Disease progression during follow-up**	
No	27 (69.2%)
Yes	12 (30.8%)

Abbreviations: ECOG, Eastern Cooperative Oncology Group; IQR, interquartile range.

**Table 2:** Concordance of hotspot oncogenic somatic mutations in the *KRAS*, *NRAS*, and *BRAF* genes between matched FFPE tissue and baseline plasma samples.

Patient ID	Stage	Hotspot Somatic Mutations		Comments
		FFPE (Routine PCR Testing)	Plasma (p01) (Targeted NGS Panel)	
LB_UC05	IVB	*KRAS* G12/13X	*KRAS* G12D, 21.1%	Concordant
LB_UC06	IVA	*KRAS* G12/13X	*KRAS* G12R, 0.592%	Concordant
LB_UC07	IVC	wt	wt	Concordant
LB_UC08	IVC	wt	*KRAS* L19F, 3.45%	Hotspot-concordant; rare activating *KRAS* mutation detected
LB_UC09	IVA	wt	wt	Concordant
LB_UC10	IVB	*KRAS* G12/13X	*KRAS* G12V, 43.0%	Concordant
LB_UC11	IVA	*KRAS* G12/13X	wt	Low coverage of cfDNA by consensus reads
LB_UC12	IVA	*KRAS* G12A	*KRAS* G12A, 7.61%	Concordant
LB_UC13	IVA	wt	wt	Concordant
LB_UC14	IVA	wt	wt	Concordant
LB_UC15	IVA	wt	*KRAS* A59T, 0.394%	Hotspot-concordant; rare activating *KRAS* mutation detected
LB_UC16	IVC	*BRAF* V600E	*BRAF* V600E, 6.2%	The *BRAF* V600E mutation was detected in cfDNA despite low coverage by consensus reads
LB_UC17	IVA	*KRAS* G12/13X	*KRAS* G13D, 31.8%	Concordant
LB_UC18	IVC	*KRAS* G12/13X	*KRAS* G13D, 39.0%	Concordant
LB_UC19	IVB	*KRAS* G12/13X	*KRAS* G13D, 2.68%	Concordant
LB_UC20	IVC	*KRAS* G12/13X	*KRAS* G13D, 11.1%	Concordant
LB_UC21	IVA	wt	wt	Concordant
LB_UC22	IVA	wt	wt	Concordant
LB_UC23	IVA	wt	wt	Low coverage of cfDNA by consensus reads
LB_UC24	IVC	wt	wt	Low coverage of cfDNA by consensus reads
LB_UC25	IVB	wt	wt	Concordant
LB_UC26	IVA	*NRAS* G12C	wt	The *NRAS* G12C mutation was not detected at p01 but was detected at p02 and p03 with VAFs of 1.52% and 0.36%, respectively
LB_UC27	IVB	*KRAS* G12D	*KRAS* G12D, 19.8%	Concordant
LB_UC28	IVA	wt	wt	Concordant
LB_UC29	IVB	wt	wt	Concordant
LB_UC30	IVB	*KRAS* K117X/A146X	*KRAS* A146P, 53.1%	Concordant
LB_UC31	IVA	wt	wt	Concordant
LB_UC32	IVA	*KRAS* G12V	*KRAS* G12V, 3.66%	Concordant
LB_UC33	IVC	*BRAF* V600E	*BRAF* V600E, 1.37%	Concordant
LB_UC34	IVC	wt	wt	Concordant
LB_UC35	IVA	*KRAS* G13D	*KRAS* G13D, 10.6%	Concordant
LB_UC36	IVC	wt	*KRAS* A146T, 34.5%	*KRAS* A146T was not covered by routine PCR
LB_UC37	IVA	wt	wt	Concordant
LB_UC38	IVA	*KRAS* G12A	*KRAS* G12A, 33.2%	Concordant
LB_UC39	IVA	wt	wt	Concordant
LB_UC40	IVA	wt	wt	Concordant
LB_UC41	IVC	*KRAS* G12V	*KRAS* G12V, 4.74% *KRAS* G12D, 0.549%	Concordant; additional *KRAS* mutation detected at lower VAF
LB_UC42	IVC	*KRAS* G12D	wt	False negative

Abbreviations: FFPE, formalin-fixed, paraffin-embedded tissue; PCR, polymerase chain reaction; LB, liquid biopsy; NGS, next-generation sequencing; p01, pretreatment plasma; VAF—variant allele frequency; wt, wild type. X denotes an unspecified amino acid substitution at the indicated codon, as reported by the routine tissue PCR assay.

## Data Availability

The original contributions presented in this study are included in the article/Supplementary Material. Further inquiries can be directed to the corresponding authors.

## Supplementary Materials

The following supporting information can be downloaded at https://www.mdpi.com/article/10.3390/ijms27219633/s1.

## Abbreviations

The following abbreviations are used in this manuscript:

LB	Liquid biopsy
cfDNA	Cell-free DNA
ctDNA	Circulating tumor DNA
VAF	Variant allele frequency
maxVAF	Maximum variant allele frequency
aggVAF	Aggregated variant allele frequency
UMI	Unique molecular identifier
mCRC	Metastatic colorectal cancer
IQR	Interquartile range
NGS	Next-generation sequencing
PCR	Polymerase chain reaction
FFPE	Formalin-fixed, paraffin-embedded
FOLFOX	Folinic acid, fluorouracil, and oxaliplatin
CI	Confidence interval
PPV	Positive predictive value
NPV	Negative predictive value
FDR	False discovery rate
RECIST	Response Evaluation Criteria in Solid Tumors
ECOG	Eastern Cooperative Oncology Group
