# Supplementary material for: Serial Tumor-Agnostic Plasma Profiling with a Compact NGS Panel During First-Line Therapy for Newly Diagnosed Metastatic Colorectal Cancer
Source: Int J Mol Sci. 2026 Oct 28;27(21):9633. doi: 10.3390/ijms27219633 (PMC13763268; doi:10.3390/ijms27219633)
Supplement: Supplementary file 1 [file ijms-27-09633-s001.zip › IJMS_LB_mCRC_Supplementary Materials_20261019b.pdf]

Supplementary Materials for

Serial Tumor-Agnostic Plasma Profiling with a Compact NGS Panel During First-Line Therapy for Newly Diagnosed Metastatic Colorectal Cancer

Galina Zakharova*, Oleg Zauzolkov, Anna Khristichenko, Vladimir Lyadov, Ivan Gaziev, Nina Shaban, Anna Emelyanova, Marina Sekacheva, Marina Lyadova, Anton Buzdin*

* Correspondence: zakharova_g_s@staff.sechenov.ru, buzdin_a_a@staff.sechenov.ru

This PDF file includes:

Figures S1 to S15

Table S1

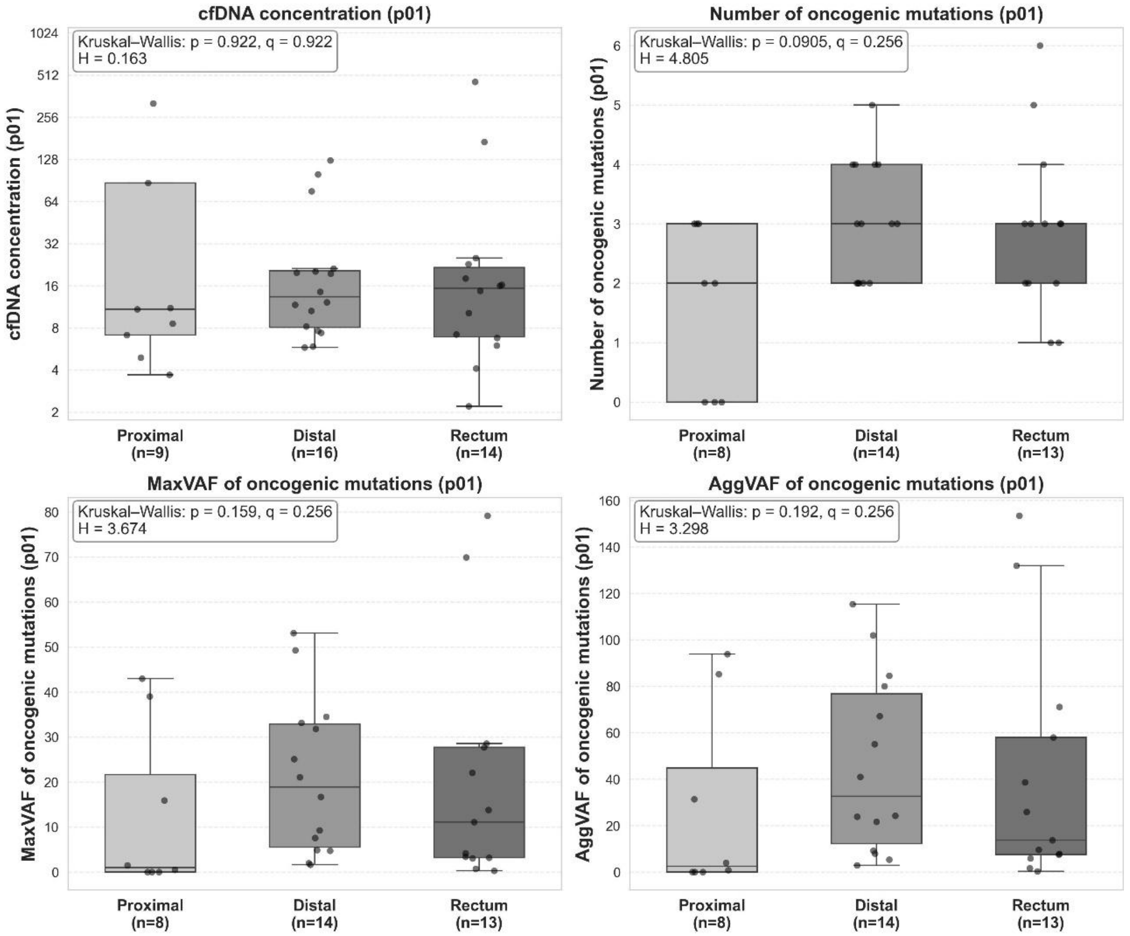

Figure S1. Association of liquid biopsy parameters with primary tumor localization in patients with metastatic colorectal cancer before treatment (p01). MaxVAF, maximum variant allele frequency of detected in cfDNA oncogenic somatic mutations; AggVAF, aggregated variant allele frequencies of all detected in cfDNA oncogenic somatic mutations.

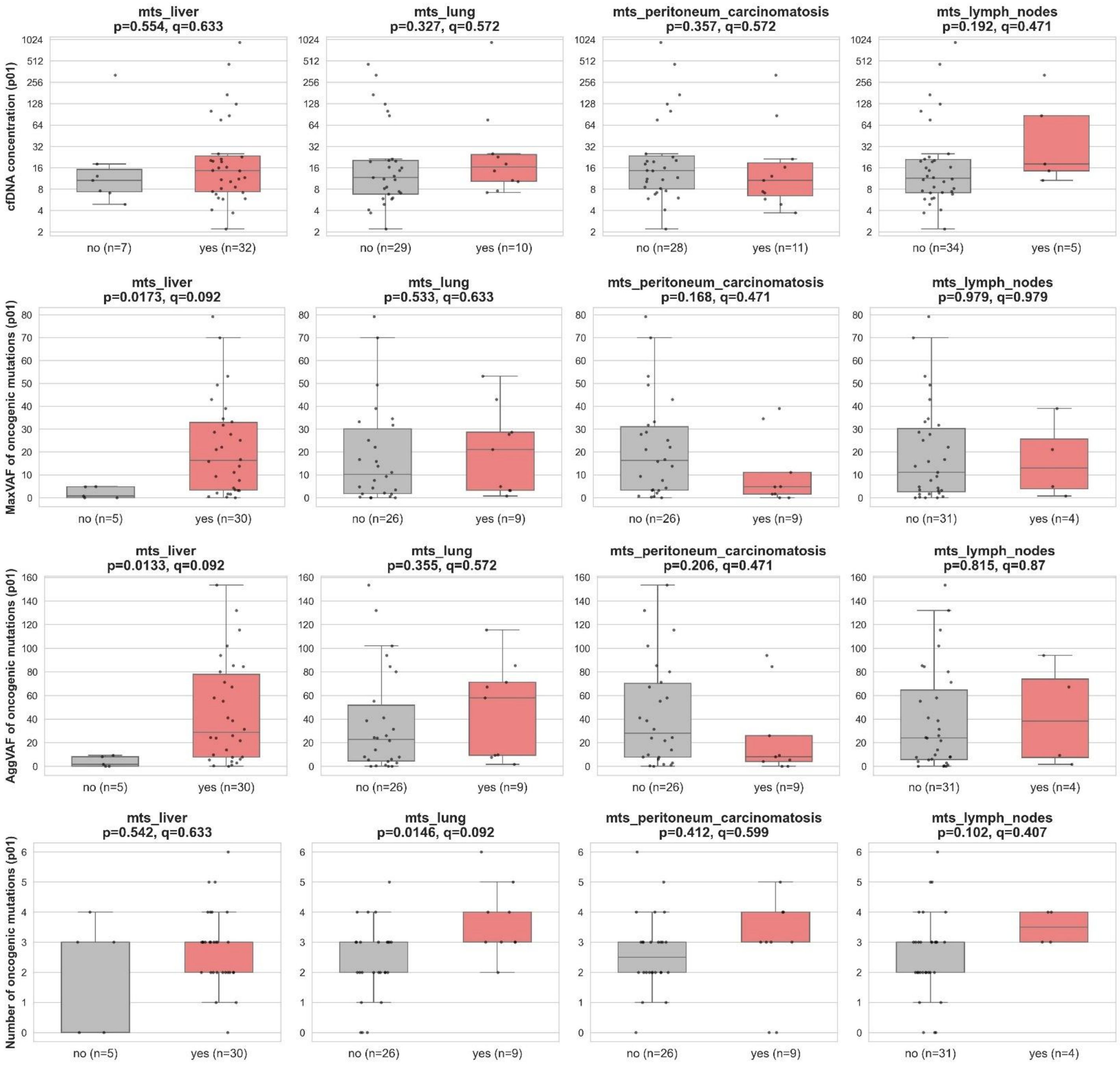

Figure S2. Association of liquid biopsy parameters with individual metastatic sites in patients with metastatic colorectal cancer before treatment (p01). MaxVAF – maximum variant allele frequency of detected in cfDNA oncogenic somatic mutations; AggVAF – aggregated variant allele frequencies of all detected in cfDNA oncogenic somatic mutations.

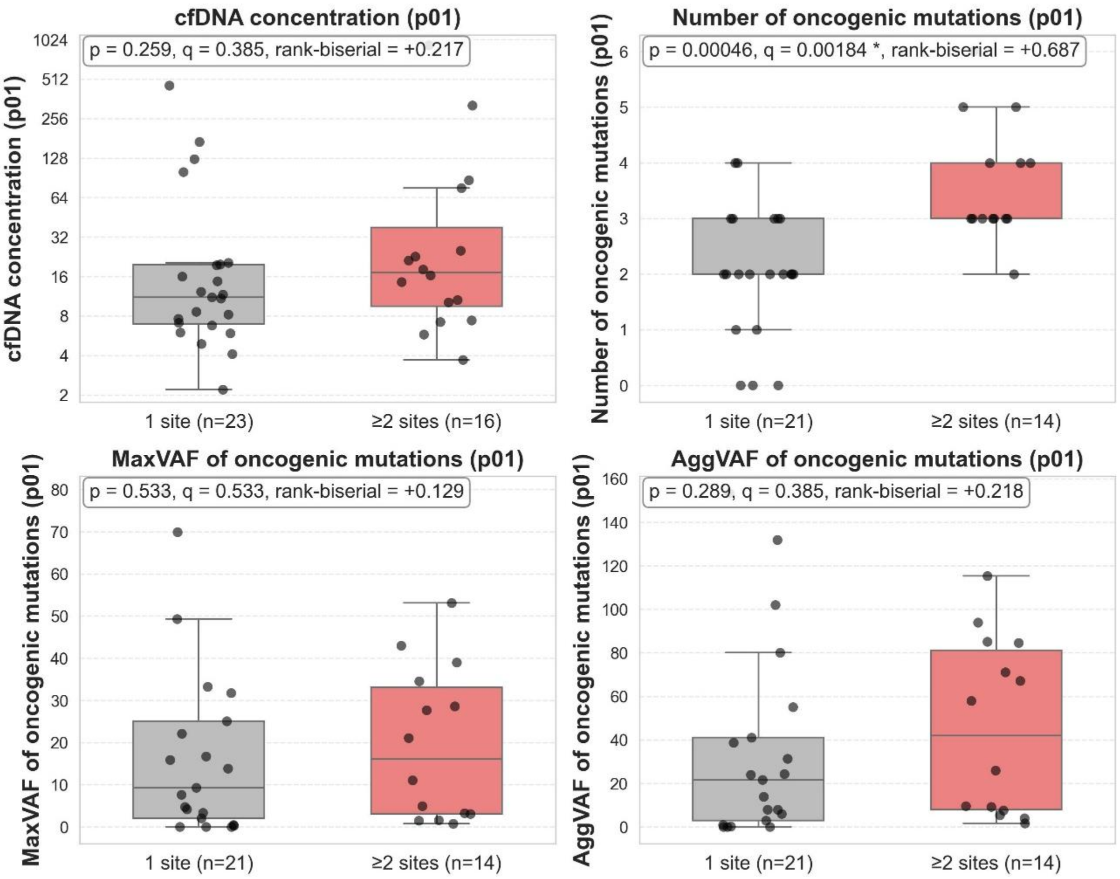

Figure S3. Association of liquid biopsy parameters with number of metastatic sites in patients with metastatic colorectal cancer before treatment (p01). MaxVAF, maximum variant allele frequency of detected in cfDNA oncogenic somatic mutations; AggVAF, aggregated variant allele frequencies of all detected in cfDNA oncogenic somatic mutations.

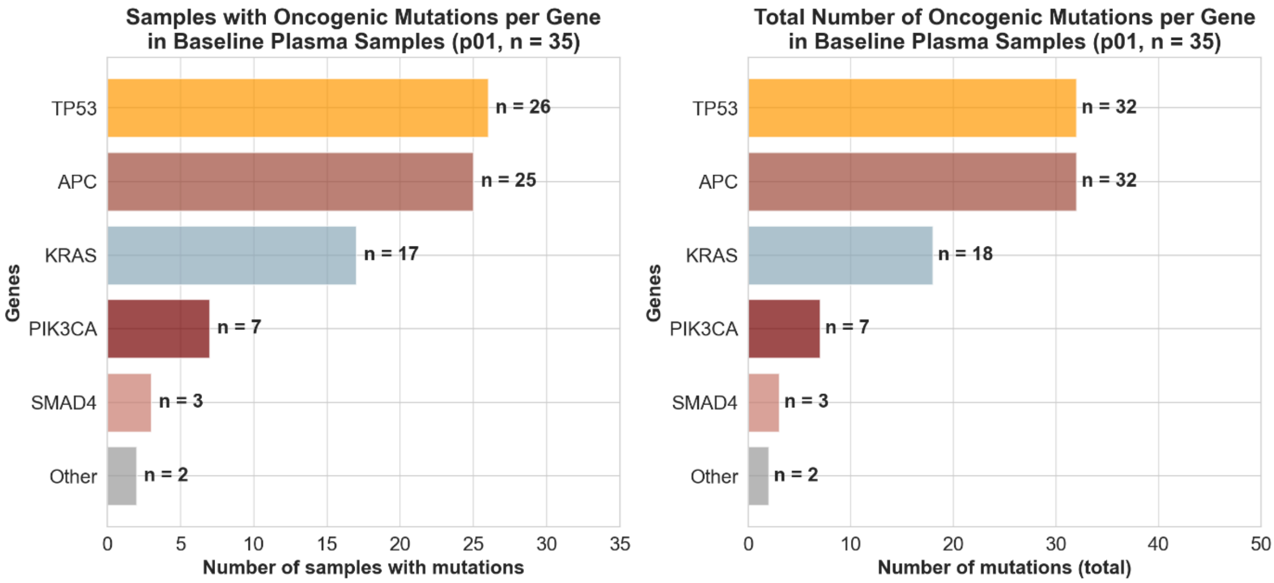

Figure S4. Profile of detected oncogenic somatic mutations in pretreatment plasma samples (p01) from mCRC patients. The left panel shows the number of samples with oncogenic mutations in each gene, and the right panel shows the total number of oncogenic mutations detected in each gene.

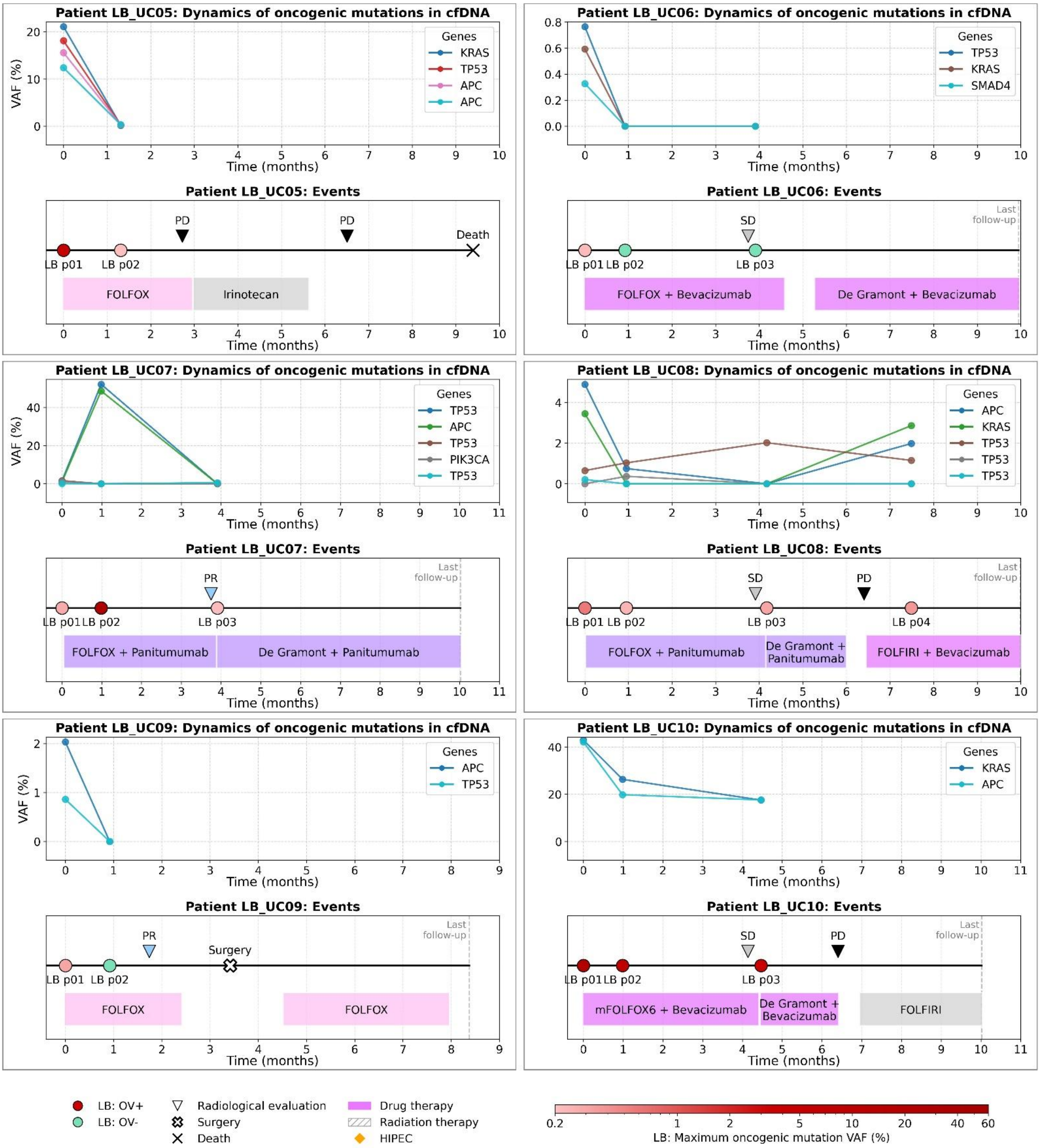

Figure S5. Longitudinal dynamics of oncogenic somatic mutation variant allele frequencies (VAFs) in cfDNA and treatment course and radiological response in individual patients with mCRC. LB, liquid biopsy; ctDNA, circulating tumor DNA; HIPEC, hyperthermic intraperitoneal chemotherapy; CR, complete response; PR, partial response; SD, stable disease; PD, progressive disease.

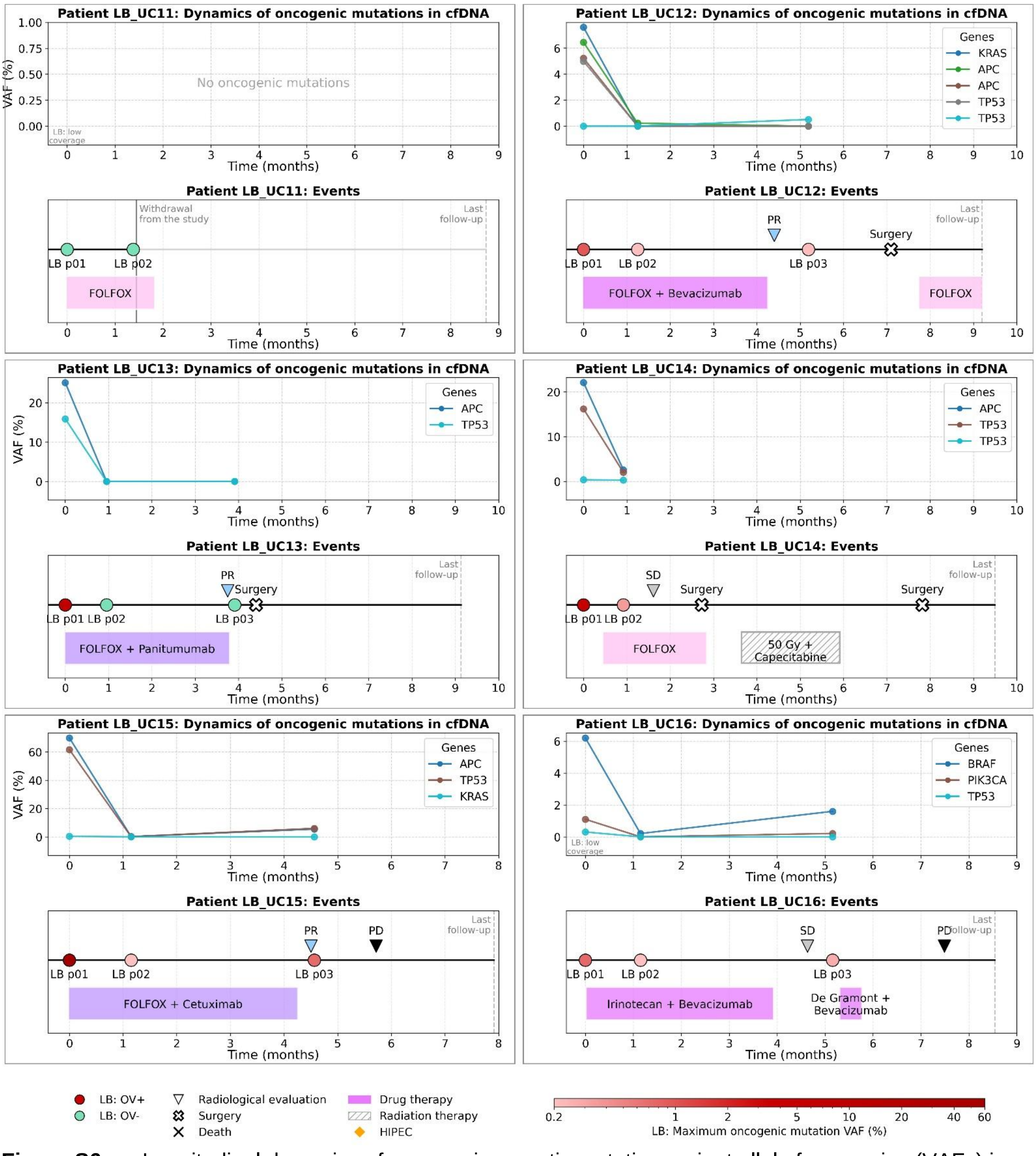

Figure S6. Longitudinal dynamics of oncogenic somatic mutation variant allele frequencies (VAFs) in cfDNA and treatment course and radiological response in individual patients with mCRC. LB, liquid biopsy; ctDNA, circulating tumor DNA; HIPEC, hyperthermic intraperitoneal chemotherapy; CR, complete response; PR, partial response; SD, stable disease; PD, progressive disease.

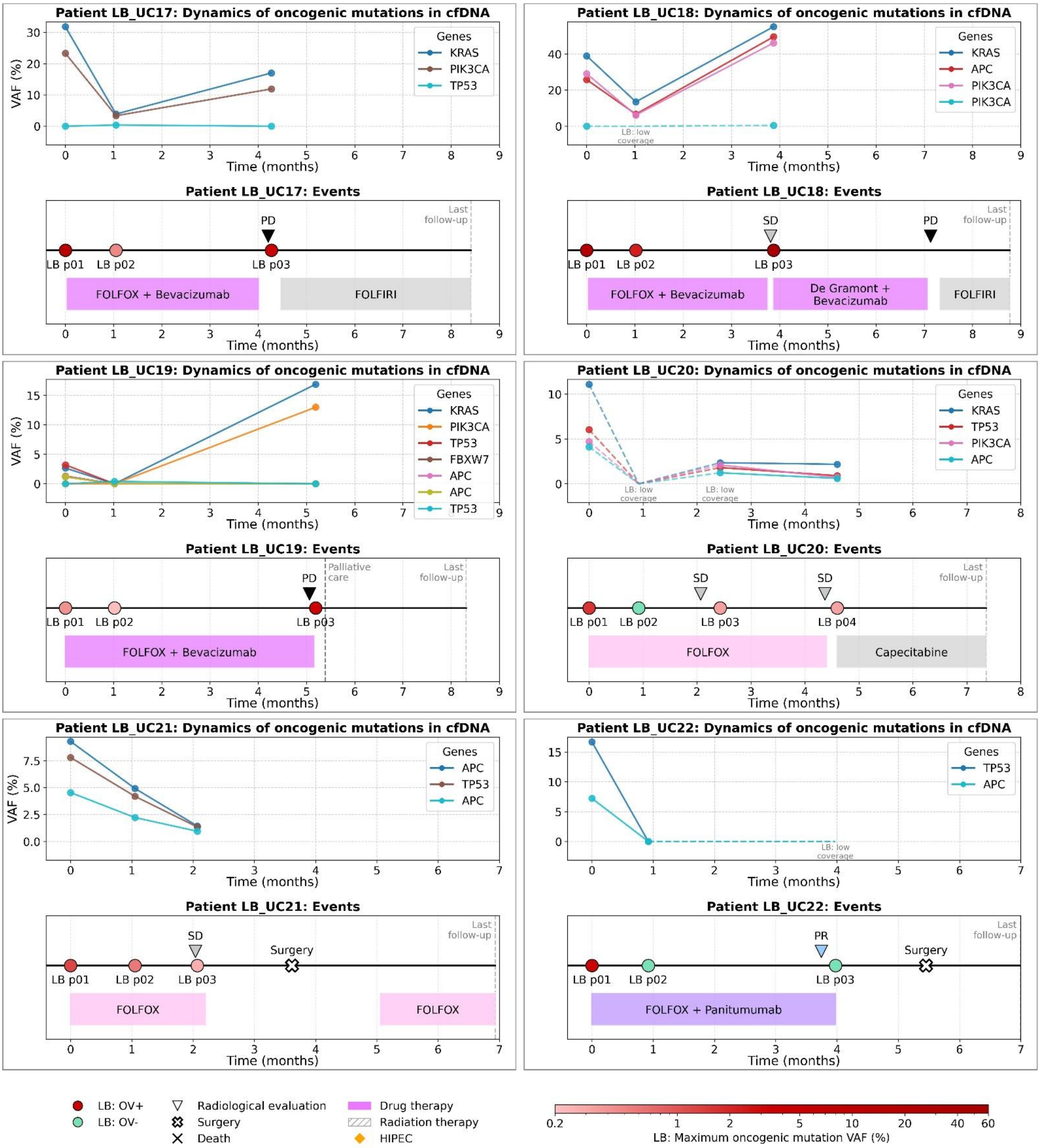

Figure S7. Longitudinal dynamics of oncogenic somatic mutation variant allele frequencies (VAFs) in cfDNA and treatment course and radiological response in individual patients with mCRC. LB, liquid biopsy; ctDNA, circulating tumor DNA; HIPEC, hyperthermic intraperitoneal chemotherapy; CR, complete response; PR, partial response; SD, stable disease; PD, progressive disease.

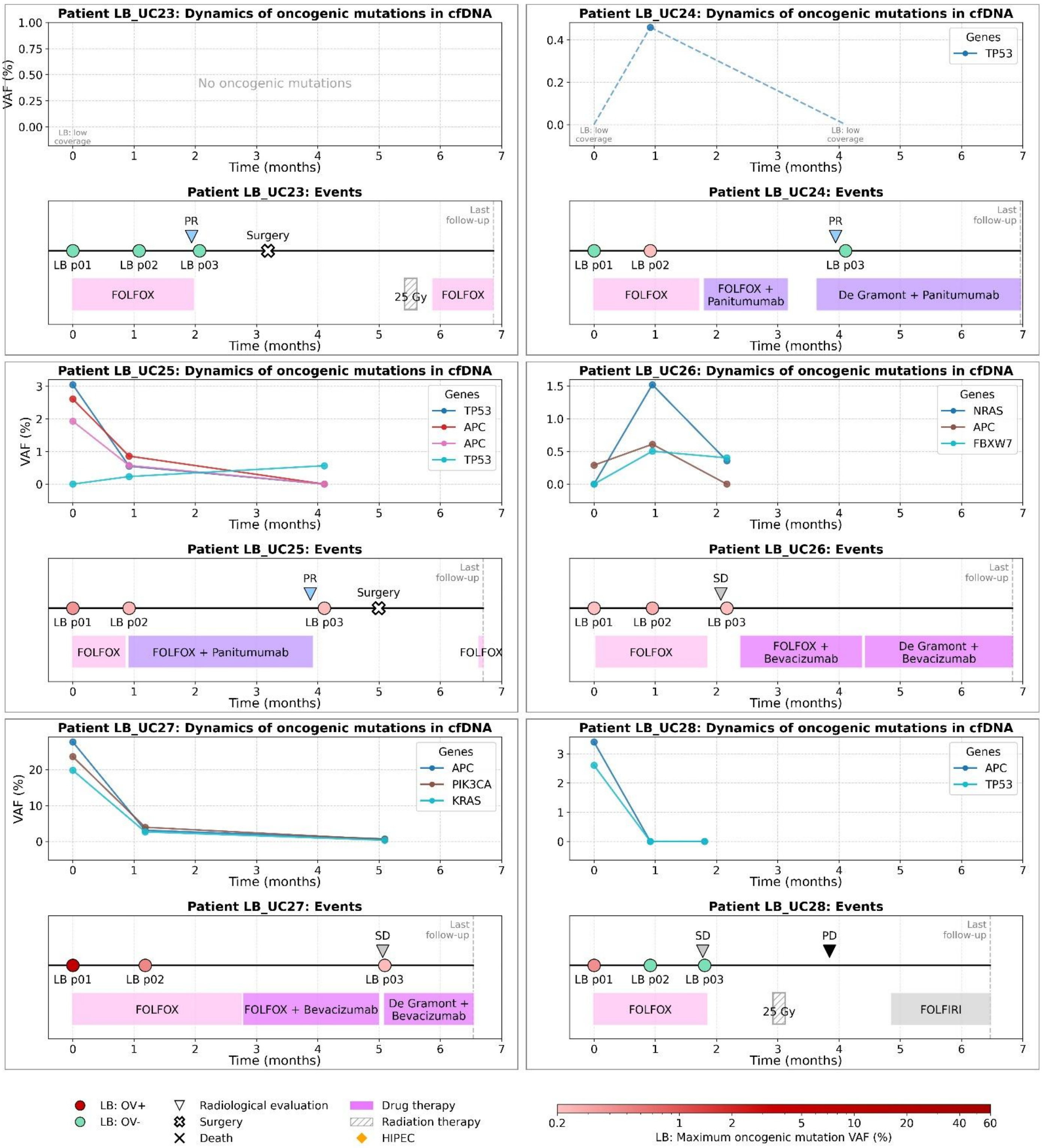

Figure S8. Longitudinal dynamics of oncogenic somatic mutation variant allele frequencies (VAFs) in cfDNA and treatment course and radiological response in individual patients with mCRC. LB, liquid biopsy; ctDNA, circulating tumor DNA; HIPEC, hyperthermic intraperitoneal chemotherapy; CR, complete response; PR, partial response; SD, stable disease; PD, progressive disease.

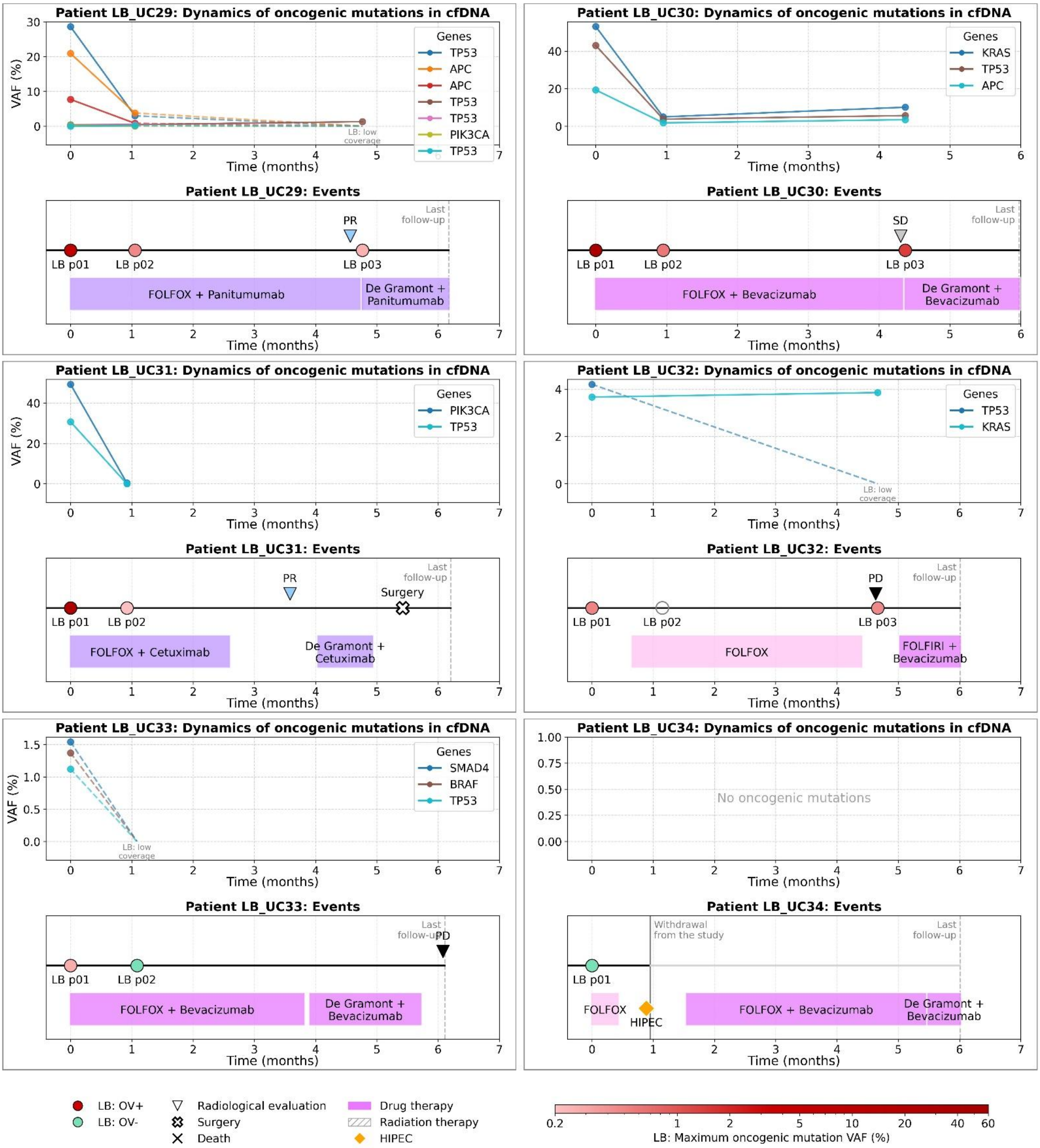

Figure S9. Longitudinal dynamics of oncogenic somatic mutation variant allele frequencies (VAFs) in cfDNA and treatment course and radiological response in individual patients with mCRC. LB, liquid biopsy; ctDNA, circulating tumor DNA; HIPEC, hyperthermic intraperitoneal chemotherapy; CR, complete response; PR, partial response; SD, stable disease; PD, progressive disease.

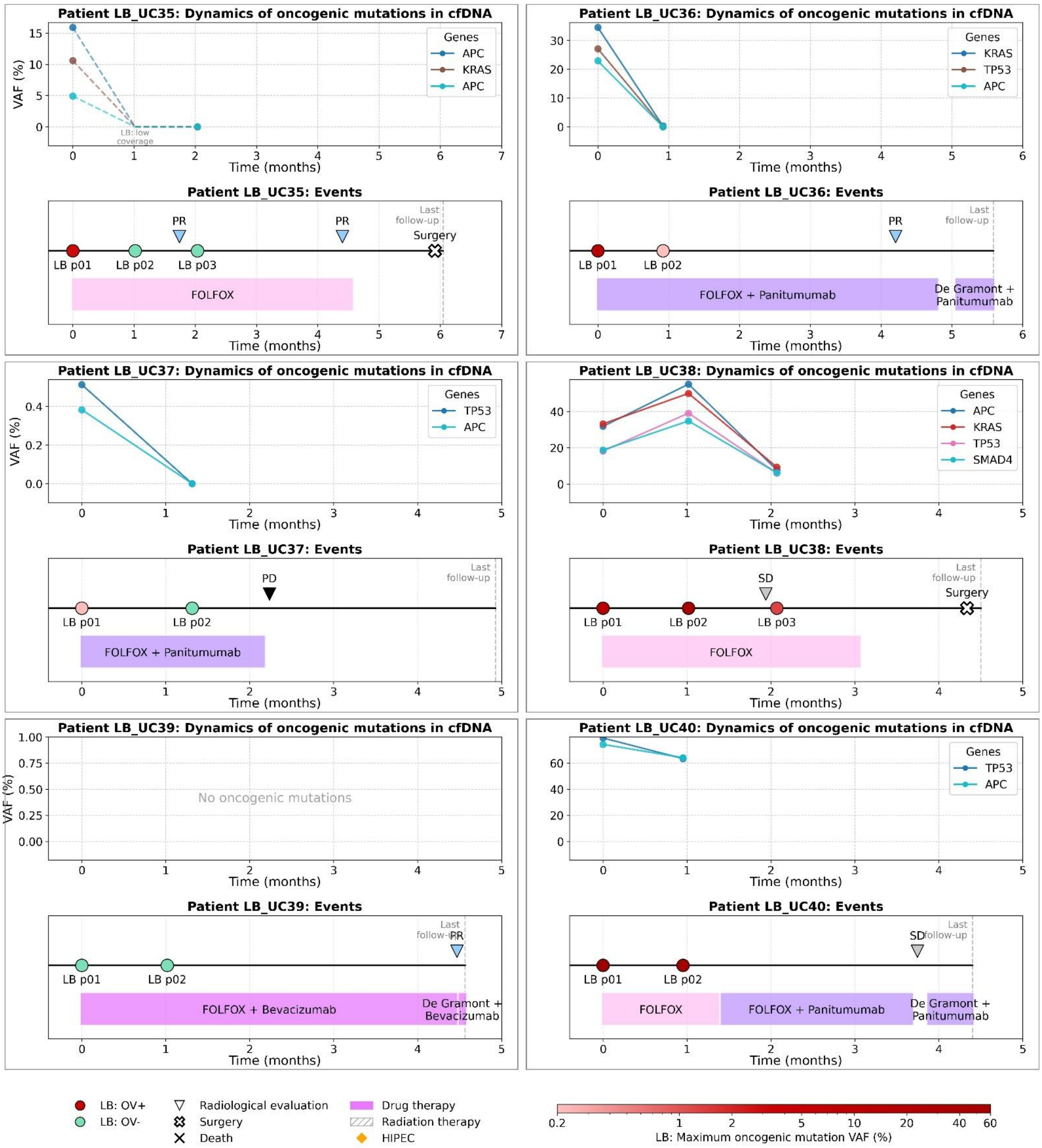

Figure S10. Longitudinal dynamics of oncogenic somatic mutation variant allele frequencies (VAFs) in cfDNA and treatment course and radiological response in individual patients with mCRC. LB, liquid biopsy; ctDNA, circulating tumor DNA; HIPEC, hyperthermic intraperitoneal chemotherapy; CR, complete response; PR, partial response; SD, stable disease; PD, progressive disease.

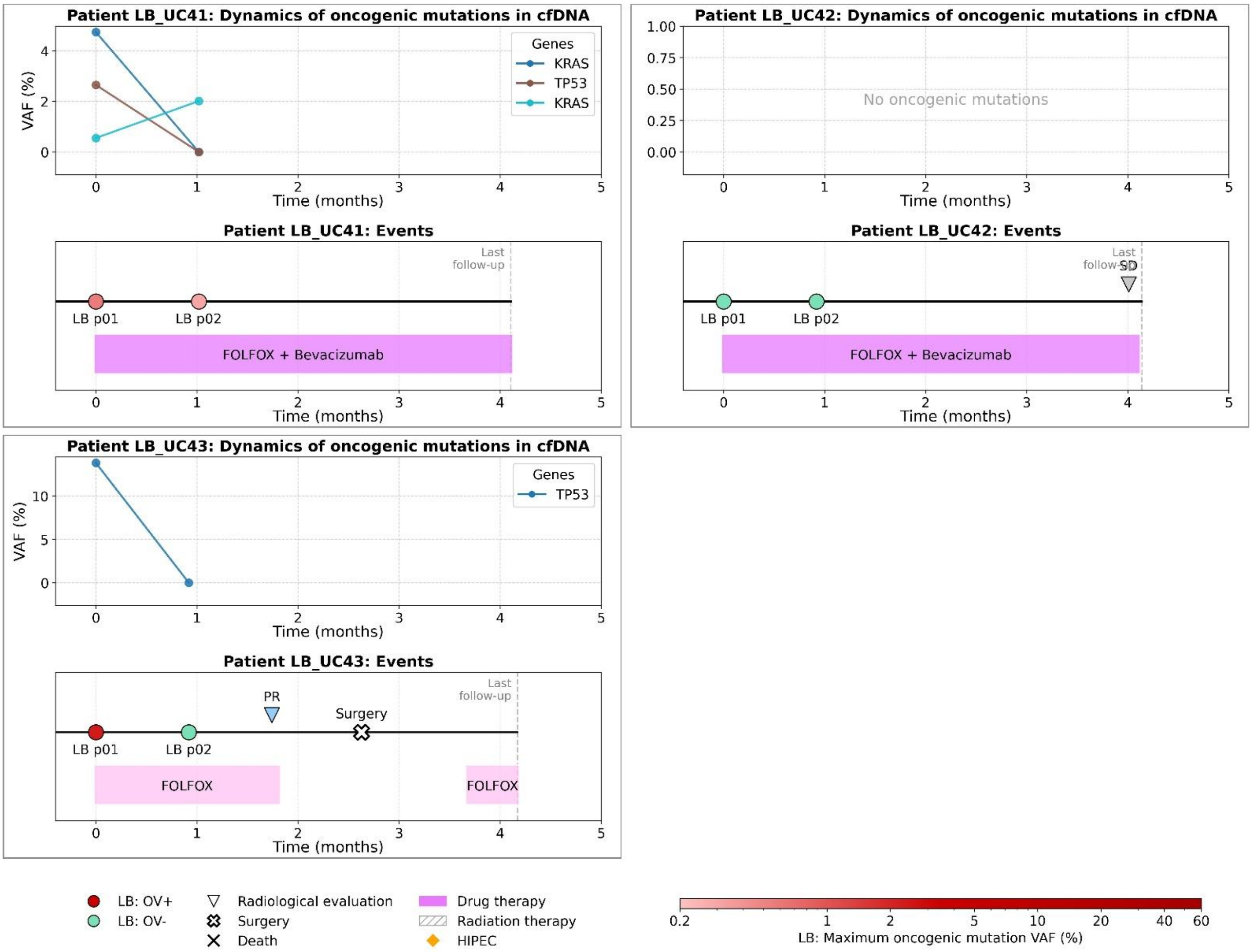

Figure S11. Longitudinal dynamics of oncogenic somatic mutation variant allele frequencies (VAFs) in cfDNA and treatment course and radiological response in individual patients with mCRC. LB, liquid biopsy; ctDNA, circulating tumor DNA; HIPEC, hyperthermic intraperitoneal chemotherapy; CR, complete response; PR, partial response; SD, stable disease; PD, progressive disease.

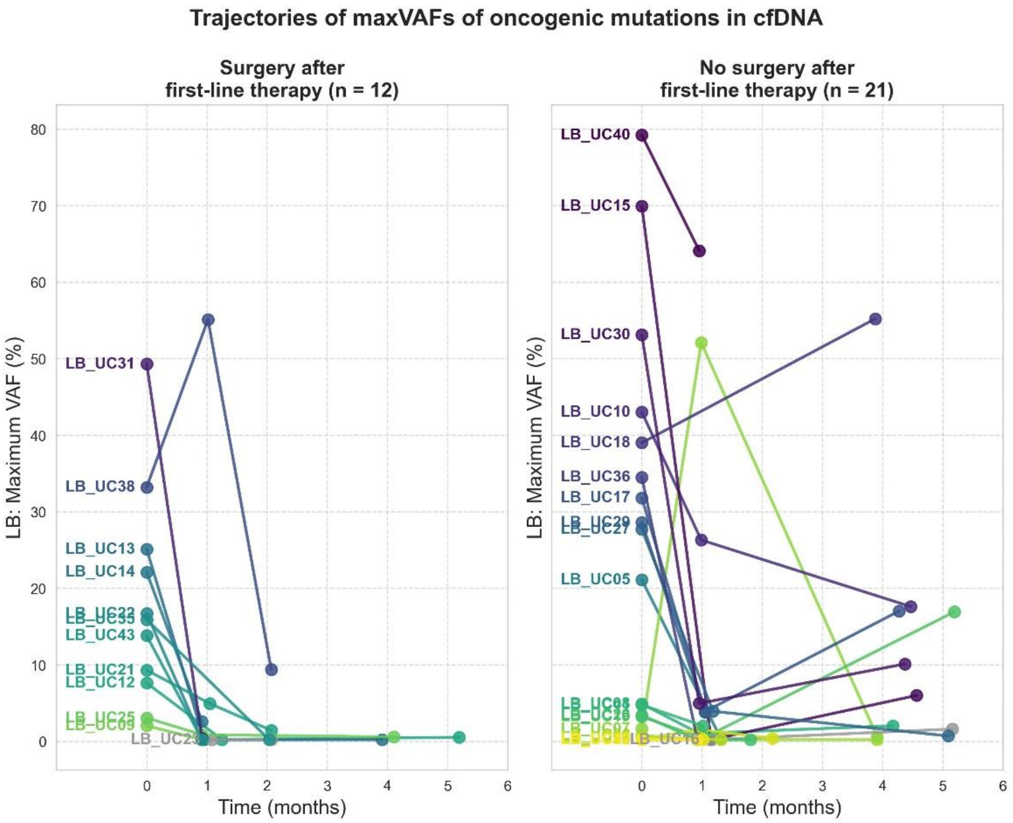

Figure S12. Trajectories of maximum variant allele frequencies (maxVAFs) of oncogenic somatic mutations in plasma during first-line systemic therapy. Patients who underwent surgery after first-line systemic therapy are shown on the left, whereas patients who did not undergo surgery are shown on the right. cfDNA, cell-free DNA; LB, liquid biopsy.

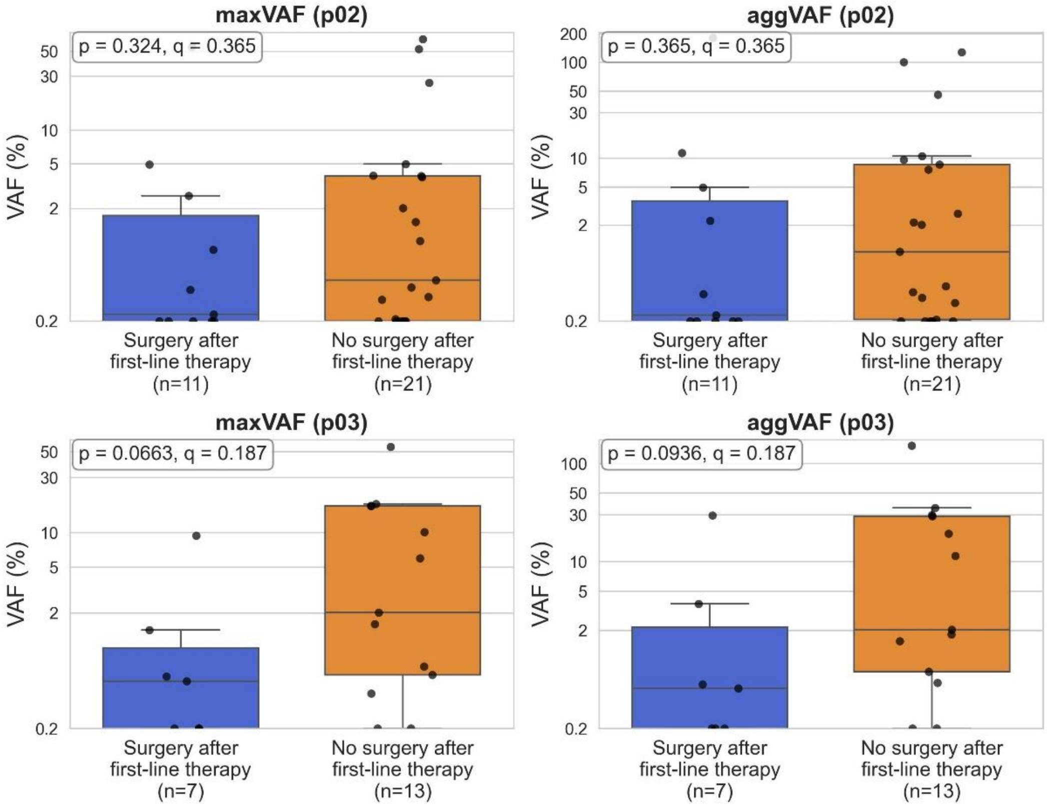

Figure S13. Comparison of plasma ctDNA levels between patients with mCRC undergoing surgery and those not undergoing surgery after first-line therapy. Boxplots show the distribution of maximum variant allele frequency (maxVAF) and aggregated variant allele frequency (aggVAF) after two cycles of systemic therapy (p02) and approximately at the time of the first radiological assessment (p03).

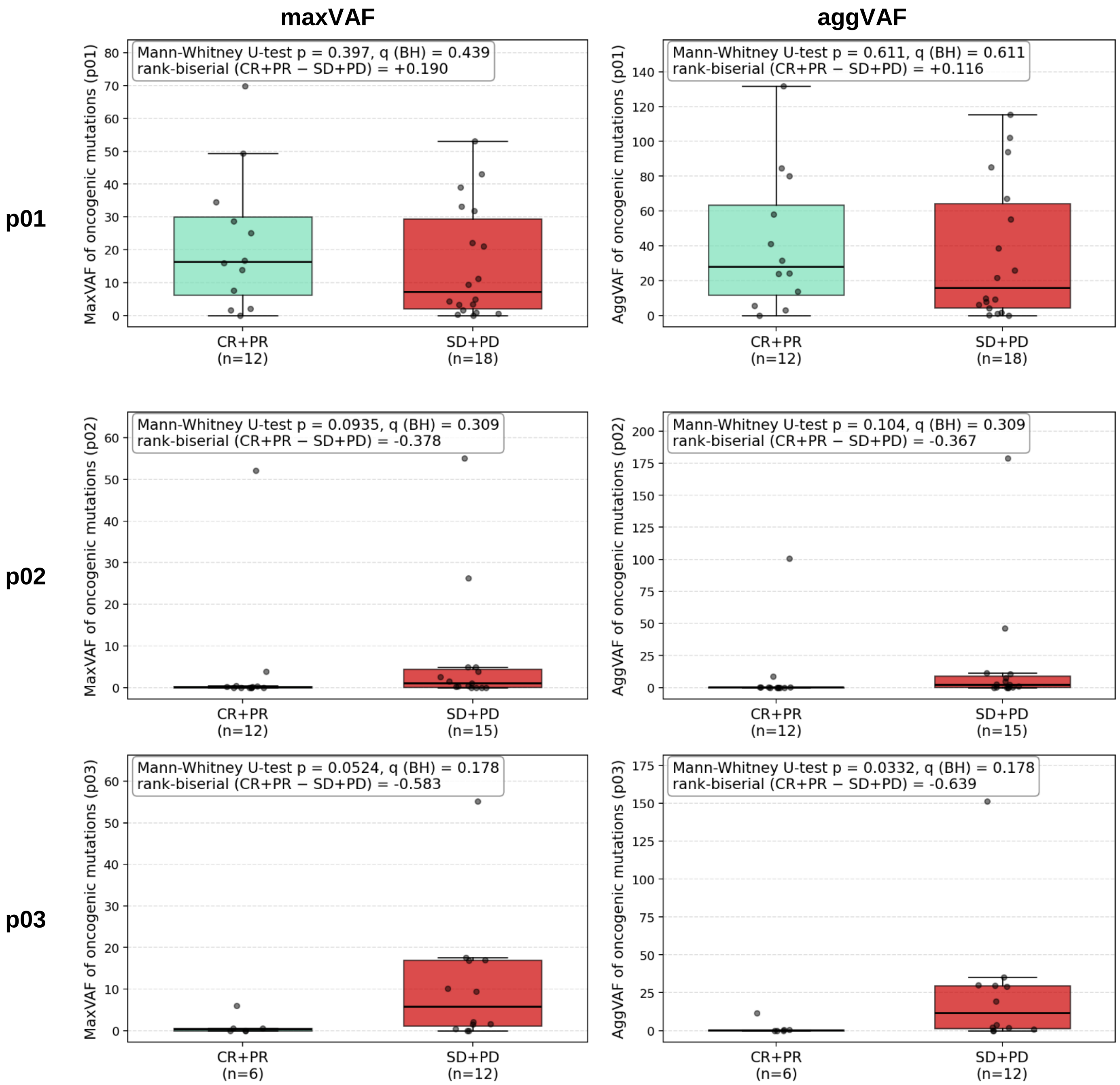

Figure S14. Comparison of plasma ctDNA levels between responders and non-responders according to the first radiological assessment using RECIST 1.1 criteria. Responders included patients with complete response (CR) or partial response (PR), whereas non-responders included patients with stable disease (SD) or progressive disease (PD). Boxplots show the distribution of maximum variant allele frequency (maxVAF) and aggregated variant allele frequency (aggVAF) before treatment (p01), after two cycles of systemic therapy (p02) and approximately at the time of the first radiological assessment (p03).

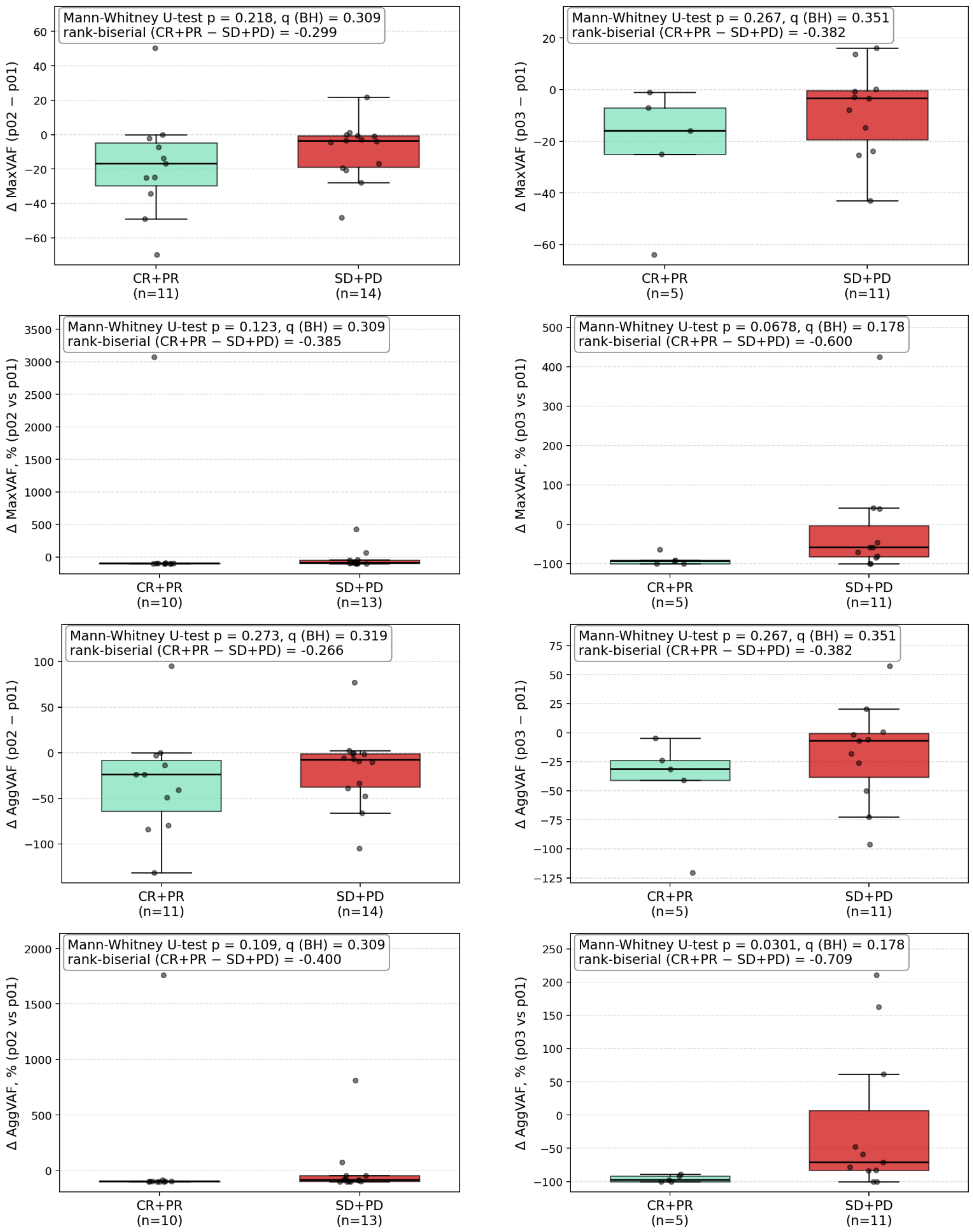

Figure S15. Comparison of changes in plasma VAFs between responders and non-responders according to the first radiological assessment using RECIST 1.1 criteria. Responders included patients with complete response (CR) or partial response (PR), whereas non-responders included patients with stable disease (SD) or progressive disease (PD). Changes in maximum variant allele frequency (ΔmaxVAF) and aggregated variant allele frequency (ΔaggVAF; sum of VAFs of all oncogenic somatic variants) are shown for p02 and p03 relative to baseline (p01), both as absolute changes and as percentage changes relative to baseline. Blood samples were collected before treatment (p01), after two cycles of systemic therapy (p02), and approximately at the time of the first radiological assessment (p03).

Table S1. Genes analyzed by targeted NGS.

Gene	Region Analyzed	Gene Category
KRAS	Specific exons (Including exons 2, 3, 4)	Oncogene
NRAS	Specific exons (Including exons 2, 3)	Oncogene
BRAF	Specific exons (Including exon 15)	Oncogene
APC	Specific exons	Tumor suppressor gene
TP53	Specific exons	Tumor suppressor gene
PIK3CA	Specific exons	Oncogene
FBXW7	Specific exons	Tumor suppressor gene
SMAD2	Specific exons	Tumor suppressor gene
SMAD4	Specific exons	Tumor suppressor gene
CTNNB1	Specific exons	Oncogene
